# Food as Friend or Foe: A Decadal Narrative Review of Dietary Patterns as Determinants of Gastrointestinal Pathophysiology and Clinical Outcomes (2015–2025)

**DOI:** 10.3390/ijms27062837

**Published:** 2026-03-20

**Authors:** Lavinia Cristina Moleriu, Raluca Lupusoru, Ruxandra-Cristina Marin, Călin Muntean, Teodora Piroș, Daliborca Cristina Vlad, Andrei Luca Dumitrașcu, Victor Dumitrașcu

**Affiliations:** 1 Department III, Functional Science, Discipline of Medical Informatics and Biostatistics, “Victor Babes” University of Medicine and Pharmacy, 300041 Timisoara, Romania; moleriu.lavinia@umft.ro (L.C.M.); raluca.lupusoru@umft.ro (R.L.); teodora.piros@student.umft.ro (T.P.); 2 Doctoral School of Medicine, “Victor Babes” University of Medicine and Pharmacy, 300041 Timisoara, Romania; andrei.dumitrascu@umft.ro; 3 Center for Modeling Biological Systems and Data Analysis, “Victor Babes” University of Medicine and Pharmacy, 300041 Timisoara, Romania; 4 Gastroenterology and Hepatology Clinic, County Emergency Hospital “Pius Brinzeu”, 300723 Timisoara, Romania; 5 Discipline of Pharmacology, Clinical Pharmacology and Pharmacotherapy, “Carol Davila” University of Medicine and Pharmacy, 050474 Bucharest, Romania; 6 Doctoral School of Biological and Biomedical Sciences, University of Oradea, 410087 Oradea, Romania; 7 Department IV, Biochemistry and Pharmacology, Discipline of Pharmacology, “Victor Babes” University of Medicine and Pharmacy, 300041 Timisoara, Romania; vlad.daliborca@umft.ro (D.C.V.); dumitrascu.victor@umft.ro (V.D.)

**Keywords:** dietary patterns, gut microbiome, inflammatory bowel disease, irritable bowel syndrome, celiac disease, Mediterranean diet, low-FODMAP diet, probiotics, prebiotics, precision nutrition, short-chain fatty acids, gastrointestinal health

## Abstract

Diet is a major modifiable determinant of gastrointestinal (GI) health, influencing disease risk and progression through coordinated effects on the gut microbiome, immune regulation, epithelial barrier integrity, oxidative balance, and epigenetic mechanisms. This narrative review synthesizes epidemiological, mechanistic, and clinical evidence from the past decade to examine bidirectional relationships between dietary patterns and seven common GI disorders: celiac disease, irritable bowel syndrome (IBS), Crohn’s disease, ulcerative colitis, *Helicobacter pylori*-associated gastritis, peptic ulcer disease, and lactose intolerance. Western dietary patterns, characterized by high intake of ultra-processed foods and saturated fats and low fiber consumption, are consistently associated with microbial dysbiosis, impaired barrier function, and enhanced inflammatory signaling. In contrast, Mediterranean and plant-forward dietary patterns show protective associations across multiple GI conditions. Mechanistically, diet influences GI pathophysiology largely through microbiome-derived metabolites, particularly short-chain fatty acids, which regulate epithelial homeostasis, immune tolerance, and inflammatory pathways. Condition-specific dietary strategies remain clinically important. Gluten exclusion is essential in celiac disease, low- fermentable oligosaccharides, disaccharides, monosaccharides and polyols (FODMAP) approaches provide evidence-based symptom control in IBS, and exclusive enteral nutrition or targeted exclusion diets support remission induction in Crohn’s disease. Selected probiotics and emerging postbiotics may provide adjunctive benefits in specific contexts. Despite growing evidence, dietary research remains limited by methodological heterogeneity and interindividual variability. Precision nutrition approaches integrating microbiome profiling and artificial intelligence represent a promising translational direction. Overall, dietary patterns—rather than isolated nutrients—form the foundation of GI dietary therapy.

## 1. Introduction

Over the last decade, gastroenterology has moved from treating diet as a contextual lifestyle variable to recognizing it as a mechanistically grounded, clinically actionable exposure. This shift was enabled by converging evidence from multi-omics technologies, large prospective cohorts, and mechanistic studies demonstrating that dietary constituents can reshape epithelial signaling, immune regulation, and gut microbial ecology [1,2,3]. In this updated conceptual model, diet functions both as a potential pathogenic driver and as a therapeutic tool—“friend” and “foe”—depending on dietary pattern, host susceptibility, microbiome configuration, and co-exposures.

Global health assessments underscore the clinical relevance of this transition. The Global Burden of Disease Study 2019 identified dietary risks as a leading contributor to disability-adjusted life years (DALYs), with meaningful gastrointestinal (GI) manifestations within this burden [4,5,6]. Inflammatory bowel disease (IBD) has provided one of the most persuasive epidemiologic narratives supporting dietary involvement: while incidence has stabilized in some high-prevalence Western nations, newly industrialized regions (East Asia, South America, Middle East) have shown rapid increases that parallel dietary westernization and expanding ultra-processed food consumption [5,6]. Ecological correlations between indices of ultra-processed food exposure and IBD emergence have been reported, supporting diet as a plausible upstream determinant of risk at the population level [7,8,9,10,11,12,13,14].

Methodologically, nutritional gastroenterology has also matured. Retrospective self-report instruments (FFQs, 24 h recalls) remain widely used but have increasingly been complemented by objective biomarkers of intake (e.g., plasma phospholipid fatty acids, urinary polyphenol metabolites, fecal short-chain fatty acids (SCFA) measurements), mitigating bias and improving exposure specificity [15,16]. Large prospective cohorts have strengthened inference. The United Kingdom (UK) Biobank (over 500,000 participants) enabled robust analyses of dietary patterns and incident disease while adjusting for multiple confounders [17]. In that cohort, higher adherence to the Alternate Mediterranean Diet (AMED) was associated with reduced Crohn’s disease risk (HR 0.58; 95% CI 0.42–0.80), with partial mediation via anti-inflammatory biomarker pathways [18]. Similarly, a 2025 systematic review/meta-analysis (72 cohorts; >2 million participants) reported increased Crohn’s risk with inflammatory dietary patterns and ultra-processed foods, and protection associated with fiber intake and Mediterranean adherence, while ulcerative colitis associations showed greater heterogeneity [19].

These epidemiologic and methodological advances intersect with a major conceptual revolution: the microbiome. Work arising from the Human Microbiome Project and subsequent initiatives reframed diet–host interactions as frequently microbiota-mediated [20,21]. The gut microbiota acts as a metabolically active “virtual organ,” transforming dietary substrates into bioactive molecules that affect barrier function, immune programming, and systemic physiology [22]. SCFAs (acetate, propionate, butyrate) are central mediators: they fuel colonocytes, reinforce barrier integrity via tight junction regulation, shape epithelial proliferation via histone deacetylases (HDAC) inhibition, and exert anti-inflammatory effects through G protein-coupled receptors (GPCR) signaling (GPR41, GPR43, GPR109A) [23,24,25]. The observation that butyrate promotes Treg differentiation through epigenetic modification at the Foxp3 locus provided a mechanistic bridge linking fiber-rich patterns to immune tolerance [26,27]. Dietary pattern shifts can reshape microbial composition rapidly (within 24 h in controlled transition) supporting the feasibility of diet as a lever for microbiome-targeted interventions [28]. Mediterranean-type diets tend to enrich SCFA producers (e.g., *Faecalibacterium prausnitzii*, *Roseburia* spp., *Bifidobacterium*), whereas Western dietary profiles favor proteolytic and sulfate-reducing organisms linked to inflammatory metabolites such as hydrogen sulfide [29,30].

Finally, the decade also introduced precision nutrition as an attempt to move beyond “one-size-fits-all” dietary recommendations [31]. Evidence for strong inter-individual variation in metabolic responses to identical foods (e.g., postprandial glycemia) and the ability of machine learning models incorporating microbiome and lifestyle variables to improve prediction encouraged translation into gastrointestinal contexts [32,33,34]. Although consumer platforms now offer microbiome-informed diet guidance, limitations remain, including training bias toward Western populations, uncertain generalizability, privacy risks, and incomplete validation of clinically meaningful outcomes [35,36]. Nevertheless, early data suggest that baseline microbiome signatures may predict response to diets such as low-fermentable, oligosaccharides, disaccharides, monosaccharides and polyols (FODMAP) in irritable bowel syndrome (IBS), and that individualized fiber prescription strategies calibrated to microbiome capacity could become relevant for maintenance strategies in IBD remission [37,38].

Moreover, the relationship between diet and GI disease is bidirectional rather than unidirectional. GI disorders can themselves reshape dietary exposure by altering food tolerance, digestion, absorption, motility, and host–microbiome interactions. In celiac disease, villous atrophy impairs nutrient absorption and may secondarily modify microbial ecology; in IBS, visceral hypersensitivity and symptom anticipation frequently narrow dietary variety and promote avoidant eating patterns; and in inflammatory bowel disease, active inflammation, bile acid malabsorption, intestinal resections, and pharmacological therapies can alter nutrient handling and tolerance to fiber, fat, and fermentable substrates. Conversely, habitual dietary patterns can influence disease initiation and progression by modulating epithelial barrier integrity, immune activation, oxidative stress, bile acid metabolism, and microbiome-derived metabolites. Diet–microbiome interactions are increasingly recognized as central mediators of these processes, with dietary substrates shaping microbial composition and metabolic output, which in turn influences host immune and epithelial responses [39,40]. At the same time, host physiology and disease states can reciprocally modify microbiome structure and metabolic activity, reinforcing the dynamic and bidirectional nature of diet–host–microbiome interactions in gastrointestinal disease [41,42].

This narrative review synthesizes evidence from 2015–2025 on how five dietary paradigms (Western, Mediterranean, low-FODMAP, plant-based, gluten-free) shape gastrointestinal pathophysiology and outcomes across celiac disease, IBS, Crohn’s disease, gastritis, peptic ulcer disease, and food intolerances (gluten and lactose). Mechanistic emphasis is placed on epithelial signaling, inflammation, oxidative stress, regulated cell death/proliferation, motility, digestion/absorption, vascular patterns, and microbiota modulation, with attention to confounding by medications (proton pomp inhibitors, histamine H2 inhibitors, and biologic therapies).

## 2. Global Epidemiology, Dietary Risk Factors, and Economic Burden

Digestive diseases account for a substantial proportion of global DALYs, with peptic ulcer disease, IBD, and functional disorders contributing meaningfully [43,44]. Over the past decade, the burden of gastrointestinal disease has shown divergent trajectories: early-industrialized nations are increasingly characterized by stable incidence but rising prevalence of chronic conditions, while newly industrialized regions show accelerating incidence, reflecting the interplay of urbanization, healthcare access, diagnostic capacity, and lifestyle transition.

For IBD specifically, a four-stage model encompassing emergence, acceleration, compounding prevalence, and eventual prevalence equilibrium helps contextualize this divergence [45]. Many Western regions are now in the compounding prevalence stage, incidence stabilizes while prevalence rises because these are chronic, non-fatal diseases, with projections suggesting that more than 1% of populations in some regions may live with IBD within the next decade [46]. In contrast, regions with historically low disease burden (parts of Asia, South America, Middle East) are experiencing rapid incidence increases that align temporally with dietary westernization, including higher consumption of ultra-processed foods, saturated fats, and refined carbohydrates alongside decreased fiber intake [47,48,49]. Global burden of disease study (GBD) analyses document increasing crude prevalence globally (e.g., 1990 to 2019) with decreasing age-standardized prevalence, consistent with demographic effects and improved survival [50]. Strong regional heterogeneity persists, with the highest age-standardized prevalence in North America and Western Europe and lower rates in East Asia and Sub-Saharan Africa, while the steepest incidence increases are observed in transitioning economies [50,51,52,53]. Prospective evidence supports diet-pattern associations more consistently for Crohn’s disease than ulcerative colitis: inflammatory dietary patterns and ultra-processed foods increase Crohn’s risk, whereas fiber and Mediterranean adherence are protective. Ulcerative colitis(UC) associations are more variable [54].

Interpretation of dietary epidemiology requires careful attention to confounding, particularly acid-suppressive medications. Proton pump inhibitors (PPIs) and histamine-2 receptor antagonists (H2 blockers) are widely used and alter gut microbiota composition in ways that can mimic or modify diet-associated microbial signatures, including reduced beneficial taxa and increased susceptibility to enteric infections [55,56,57]. Adjustment for PPIs use is inconsistently performed, and sensitivity analyses (e.g., UK Biobank) suggest modest but meaningful attenuation of diet associations after accounting for acid suppression [58]. This supports a practical recommendation for future observational and interventional designs: systematically adjust/stratify by acid suppression and evaluate effect modification.

Diet is also central to highly prevalent disorders of gut–brain interaction such as IBS, where prevalence varies sharply depending on diagnostic criteria. Rome IV (2016) introduced stricter thresholds than Rome III, lowering estimated prevalence in meta-analyses from approximately 9% (Rome III) to 4% (Rome IV), while heterogeneity remains high across studies and methodologies [59,60,61]. Female sex and psychological comorbidities are consistent correlates [60,62]. A large proportion of IBS patients report food-triggered symptoms, and low-FODMAP dietary interventions show the strongest evidence base among dietary strategies, with clinically meaningful improvement in many adherent patients [63,64,65].

Celiac disease represents a distinct paradigm: a defined immune-mediated enteropathy triggered by gluten in genetically susceptible hosts (human leukocyte antigen (HLA)-DQ2/DQ8). Meta-analyses estimate global seroprevalence 1.4% and biopsy-confirmed prevalence 0.7%, with increased risk among first-degree relatives [66,67]. Incidence appears to be increasing beyond detection effects in some datasets, implicating additional environmental modifiers (microbiota, antibiotics, early-life exposures), while the gluten-free diet remains the cornerstone of management to prevent long-term complications [68,69].

Peptic ulcer disease has declined globally, consistent with improved *H. pylori* control and changes in nonsteroidal anti-inflammatory drug (NSAID) practice, but substantial geographical disparities persist, particularly in low- and middle-income countries where *H. pylori* prevalence is higher and diagnostic/therapeutic resources are limited [70,71].

Diet may influence mucosal defense and acid dynamics and may modulate *H. pylori* virulence and persistence [72]. Chronic gastritis remains highly prevalent worldwide and forms the substrate for progression along the Correa cascade toward gastric malignancy; high salt and preserved foods increase risk, while higher fruit/vegetable intake and Mediterranean patterns appear protective in the observational literature [73,74,75,76].

Food intolerances further contribute to symptom burden and clinical uncertainty. Non-celiac gluten sensitivity (NCGS) is defined by symptom induction after gluten exposure without celiac serology/histology, but true prevalence is likely lower than self-report when evaluated by double-blind placebo-controlled challenge [77,78]. Mechanisms may include innate immune activation, increased permeability, and sensitivity to non-gluten wheat components (fructans, amylase-trypsin inhibitors) [79,80]. Lactose intolerance affects a majority of adults globally with strong ethnic variation; symptoms are dose-dependent and modified by transit time, microbiota, and visceral sensitivity [81,82,83]. It frequently overlaps with IBS and complicates dietary management, especially because lactose restriction is embedded within the broader low-FODMAP approach [64,65,84]. The key epidemiologic characteristics and diet-related associations across major gastrointestinal conditions are summarized in Table 1.

Beyond clinical burden, GI diseases impose major economic costs. In the United States, IBD-related expenditures exceed $25 billion annually, driven largely by biologic therapies, hospitalization, and long-term disease management; similar economic analyses from Europe also demonstrate substantial direct and indirect healthcare costs associated with inflammatory bowel disease [85,86]. IBS, despite being a functional disorder, generates substantial direct costs and disproportionate productivity losses through presenteeism and absenteeism [62,87,88]. Celiac disease is associated with lower direct medical costs but meaningful patient-borne dietary costs because gluten-free products are considerably more expensive than standard alternatives [89]. Emerging health economic analyses suggest that structured dietary interventions (e.g., low-FODMAP guidance for IBS) may be cost-effective compared with standard care, and that preventive dietary strategies could reduce long-term disease burden; however, comprehensive and standardized economic evaluations remain limited [90,91].

## 3. Epigenetics and Signaling Networks Linking Diet to Gastrointestinal Pathophysiology

Diet modulates GI disease risk not only through exposure patterns but also through epigenetic programming and signaling pathway activation. Epigenetic mechanisms, including deoxyribonucleic acid (DNA) methylation, histone modifications, and non-coding ribonucleic acid (RNAs), provide a molecular interface between diet-derived inputs (and microbiota-derived metabolites) and durable gene expression states [92,93]. Epigenome-Wide Association (EWAS) studies have identified differentially methylated regions in IBD enriched in genes related to immune regulation and barrier integrity, consistent with a role for epigenetic dysregulation in sustaining inflammatory phenotypes [94,95,96]. Importantly, epigenetic marks are potentially reversible, distinguishing nutritional epigenetics from fixed genetic risk and offering a rationale for diet-based modulation.

DNA methylation depends on DNA methyltransferase (DNMT) enzymes and S-adenosyl-L-methionine (SAM) as the methyl donor, linking methylation capacity to one-carbon metabolism and dietary cofactors (folate, B12, B6, B2) [97,98,99]. In IBD, folate deficiency is common due to malabsorption, increased demands, and medication interactions (e.g., methotrexate, sulfasalazine), and has been associated with aberrant methylation patterns that may favor pro-inflammatory gene expression [100,101]. Microbial contributions may further shape methylation capacity, as certain commensals synthesize folate, supporting a microbiome–methylation axis responsive to diet and pre/probiotic strategies [102]. Candidate gene methylation changes have been reported (e.g., Interferon-gamma (IFNG) hypomethylation in UC; methylation shifts in protease-activated receptor (PAR2) and other inflammatory/barrier-related loci), and more recent work has linked methylation signatures to clinically relevant outcomes such as postoperative recurrence risk in Crohn’s disease [103,104,105,106,107].

Histone modifications provide a complementary and highly diet-responsive mechanism. Butyrate is the clearest mechanistic bridge connecting dietary fiber, microbiome activity, and chromatin remodeling: as a potent histone deacetylase (HDAC) inhibitor, it increases histone acetylation at loci governing barrier integrity (tight junction proteins, mucins) and regulatory immune programs, while suppressing inflammatory transcriptional networks including NF-κB-linked outputs [108,109,110,111,112,113]. A key therapeutic implication is the Foxp3/Treg axis: butyrate promotes Foxp3 expression through increased acetylation at promoter/enhancer regions and also acts indirectly through receptors such as G protein-coupled receptors (GPR109A) to support regulatory immune phenotypes (e.g., interleukin (IL)-10) [114,115,116,117]. Reduced colonic butyrate concentrations, depleted butyrate-producing taxa, and impaired regulatory immune function in IBD reinforce the biological plausibility of fiber-rich dietary interventions and butyrate-focused therapeutic strategies [118,119].

At the signaling level, nuclear factor kappa-light-chain-enhancer of activated B cells (NF-κB) and mitogen-activated protein kinase (MAPK) pathways serve as central hubs integrating dietary inputs, microbial sensing, and cytokine cascades. Key diet-responsive signaling pathways involved in gastrointestinal inflammation and their functional outputs are outlined in Table 2.

Western dietary components, especially saturated fatty acids and dietary patterns that promote endotoxemia, can activate toll-like receptor 4/toll-like receptor 2 (TLR4/TLR2) signaling and trigger myeloid differentiation primary response 88 (MyD88)-dependent cascades culminating in nuclear factor kappa-light-chain-enhancer of activated B cells (NF-κB) nuclear translocation and inflammatory transcription (Tumor Necrosis Factor alpha (TNF-α), interleukin 1 beta (IL-1β), interleukin 6 (IL-6); cyclooxygenase-2 (COX-2); inducible nitric oxide synthase (iNOS)) [120,121,122,123,124,132,133,134]. Mitogen-activated protein kinase (MAPK) pathways (extracellular signal-regulated kinase (ERK), c-jun n-terminal kinase (JNK), p38 mitogen-activated protein kinase (p38)) interact extensively with nuclear factor kappa-light-chain-enhancer of activated B cells (NF-κB), respond to oxidative stress, and amplify inflammation through transcriptional and post-transcriptional mechanisms including stabilization of cytokine mRNA [125,126,127]. In contrast, peroxisome proliferator-activated receptor gamma (PPAR-γ) functions as a diet-responsive anti-inflammatory nuclear receptor: activation by Mediterranean pattern components (oleic acid, omega-3 polyunsaturated fatty acids (ω-3 PUFA), polyphenols) supports transrepression of nuclear factor kappa-light-chain-enhancer of activated B cells (NF-κB) and broader immunomodulatory effects; reduced peroxisome proliferator-activated receptor gamma (PPAR-γ) expression in inflammatory bowel disease (IBD) mucosa and therapeutic effects of agonists in ulcerative colitis (UC) provide pathway validation [128,129,135,136,137].

Microbiota-derived metabolites extend epigenetic and immunologic modulation beyond butyrate. Propionate and acetate influence Treg differentiation and immune programming via receptor signaling and HDAC inhibition; polyphenols modulate DNMT activity, histone acetylation states, and miRNA networks (including links to PI3K/AKT/mTOR signaling), though translation to consistent clinical benefit remains variable across compounds and contexts [138,139,140,141,142,143,144]. The principal diet–microbiome–metabolite interactions and their epigenetic and immune consequences relevant to gastrointestinal inflammation are summarized in Table 3.

These layers interact with genetic susceptibility: genome-wide association study (GWAS) have identified >240 IBD loci, yet explained heritability remains limited, consistent with strong environmental contributions. Gene–diet interactions (e.g., nucleotide-binding oligomerization domain-containing protein 2 (NOD2)/autophagy pathways and fiber responsiveness; fucosyltransferase 2 (FUT2) secretor status and microbiota configuration) may contribute to heterogeneity in diet–disease relationships and provide a rationale for precision nutrition approaches [79,145,146,147]. Emerging causal inference strategies (including epigenetic Mendelian randomization) illustrate the potential to identify epigenetic mediators of disease risk, although the cited 2025 example pertains to pollution-related epigenetic marks rather than diet directly [148].

An additional biomolecular dimension through which dietary patterns influence GI disease involves modulation of epithelial junctional integrity. The intestinal barrier is maintained by coordinated interactions among tight junction proteins (including occludin, claudins, and zonula occludens proteins) and adherents junction components such as E-cadherin and β-catenin [149]. Western-style dietary patterns rich in saturated fats, cholesterol, and oxidized cholesterol derivatives (oxysterols) have been shown to impair epithelial barrier function by promoting oxidative stress, altering membrane lipid composition, and activating inflammatory signaling pathways such as NF-κB and MAPK. These processes disrupt tight junction architecture, increase intestinal permeability, and facilitate translocation of luminal microbial products that amplify mucosal inflammation [130,131].

In contrast, bioactive compounds abundant in plant-forward and Mediterranean dietary patterns appear to exert protective effects on epithelial barrier function. Polyphenols, including flavanols such as epicatechin, have been shown to enhance expression and localization of tight junction proteins, reduce oxidative stress, and attenuate pro-inflammatory signaling within intestinal epithelial cells [150,151]. Experimental and translational studies suggest that polyphenol-rich foods can stabilize junctional complexes and improve barrier integrity partly through antioxidant effects and modulation of microbiome-derived metabolites [152,153]. These findings support the concept that dietary composition directly influences epithelial permeability and mucosal resilience, providing a mechanistic link between dietary patterns and susceptibility to inflammatory gastrointestinal disorders.

**Figure 1:**
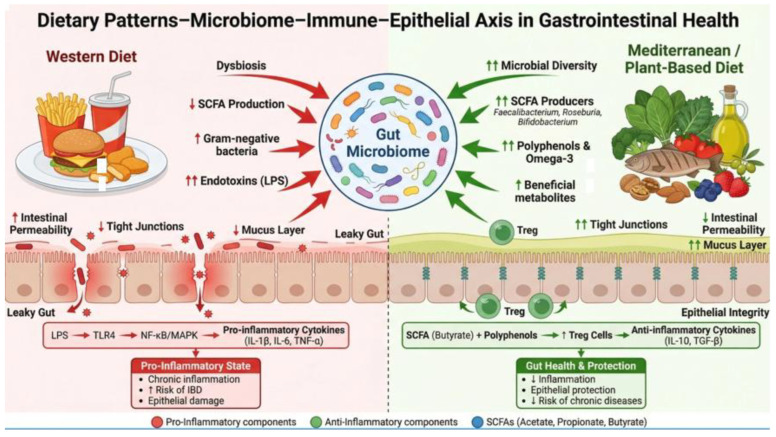
Dietary patterns–microbiome–immune–epithelial axis in GI health Schematic representation of how dietary patterns influence gastrointestinal homeostasis through interactions among the gut microbiome, epithelial barrier, and immune signaling pathways. Western-type diets promote dysbiosis, barrier disruption, and pro-inflammatory activation, whereas Mediterranean and plant-forward patterns support microbial diversity, metabolite production, and regulatory immune responses. The figure highlights diet as a central modulator of GI resilience or inflammatory susceptibility. The figure was created in Biorender, by Muntean Calin. (2026) https://BioRender.com/b8zr5jr (last accessed on 25 February 2026 for creating this image) and PowerPoint.

Dietary patterns influence gastrointestinal health through interconnected effects on the gut microbiome, epithelial barrier integrity, immune signaling, and epigenetic regulation. These mechanisms do not operate in isolation but form an integrated network linking luminal dietary exposures to mucosal inflammation and disease susceptibility. The principal epigenetic and signaling pathways through which dietary patterns modulate gastrointestinal pathophysiology are summarized in Figure 1.

Oxidative stress is a convergent mechanism. Western dietary exposures promote reactive oxygen species (ROS) via mitochondrial dysfunction, nicotinamide adenine dinucleotide phosphate (NADPH) oxidase activation, and advanced glycation end-product formation (AGE) formation, activating redox-sensitive inflammatory pathways (NF-κB, MAPK) and inflammasome signaling [154]. Systemic inflammatory biomarkers that integrate nutritional and inflammatory status, such as composite indices based on C-reactive protein and albumin, have been explored as prognostic indicators in gastrointestinal diseases, highlighting the clinical relevance of systemic inflammatory burden [155]. Saturated fat oxidation can generate higher oxidative burden than unsaturated fats; depleted glutathione pools compromise antioxidant defenses, whereas Mediterranean patterns support redox resilience through antioxidant micronutrients and substrate availability for glutathione synthesis [156,157]. NLRP3 inflammasome activation reflects a key crossroads where metabolic stress can drive pathology (e.g., ceramide and mitochondrial ROS pathways), but epithelial inflammasome signaling can also support protective IL-18 responses under some conditions, emphasizing context-dependent effects [158,159].

## 4. Characterization of Dietary Patterns: Definitions, Components, and Biological Profiles

**Figure 2:**
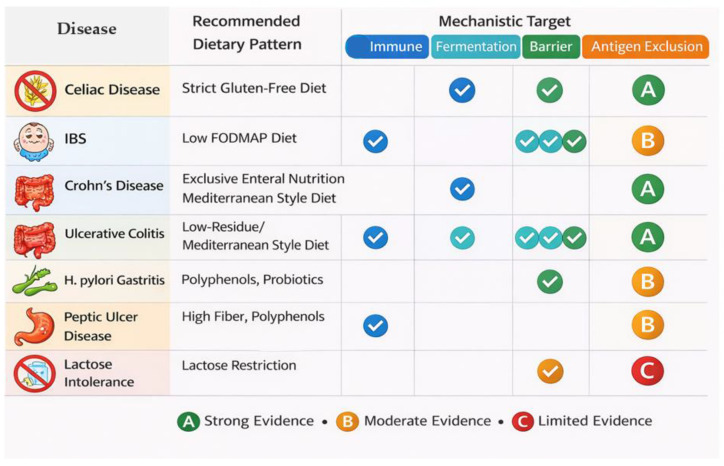
Condition-specific dietary modulation across major gastrointestinal disorders. Conceptual overview linking major GI conditions with their principal dietary strategies and dominant biological targets. Exclusion diets address causal triggers (e.g., gluten in celiac disease), while low-FODMAP approaches reduce fermentative load in IBS. Mediterranean-style patterns emphasize anti-inflammatory and microbiome-supportive effects in inflammatory bowel diseases, and adjunctive dietary measures support mucosal defense in other conditions. The figure underscores that dietary therapy is mechanism-based and disease-specific. The figure was created using BioRender.com and PowerPoint.

Dietary patterns relevant to GI health can be positioned along a continuum ranging from predominantly pro-inflammatory, barrier-disruptive exposures (typified by the Western dietary pattern) to anti-inflammatory and microbiome-supportive patterns (such as Mediterranean and plant-forward diets), alongside targeted therapeutic diets designed to address specific mechanisms, including the low-FODMAP diet for fermentative load reduction and the gluten-free diet for immune-mediated gluten intolerance. These patterns differ not only in macronutrient composition but also in degree of processing, additive exposure, fermentable substrate availability, and bioactive compound density. Together, these features influence microbial metabolism, epithelial signaling pathways, bile acid profiles, and mucosal immune tone. Figure 2 provides a conceptual overview linking major GI diseases with the dietary strategies most commonly applied to modify these biological pathways.

### 4.1. Western Dietary Pattern (Ultra-Processing, Additive Exposure, and Pro-Inflammatory Signaling)

The Western pattern is characterized by high intake of ultra-processed foods (UPFs), refined carbohydrates, saturated/trans fats, red/processed meats, sodium, and added sugars, with low intake of fiber-rich plant foods [160,161]. UPFs are operationalized via the NOVA classification, with Group 4 defined by industrial formulations containing uncommon ingredients in home cooking and additives designed to enhance palatability and shelf-life [162]. Epidemiologically, UPFs may contribute 50–60% of total energy intake in highly industrialized settings and are increasing globally [163], and recent syntheses connect this exposure to IBD risk via microbiome disruption, permeability, and innate immune activation [164].

Biologically, Western dietary exposure promotes a characteristic pattern of dysbiosis marked by reduced microbial diversity and depletion of barrier-supportive, short-chain fatty acid (SCFA)-producing taxa (e.g., *Akkermansia muciniphila*, *Faecalibacterium prausnitzii*), alongside enrichment of pathobionts such as *Bilophila wadsworthia*, members of the Enterobacteriaceae family, and sulfate-reducing bacteria [165,166,167]. Functionally, these compositional shifts are associated with diminished SCFA generation, compromised mucus layer integrity, and increased epithelial permeability. Postprandial “metabolic endotoxemia” provides a key mechanistic link: high-fat meals can enhance lipopolysaccharide (LPS) translocation and elevate circulating endotoxin levels, activating TLR4–MyD88 signaling and downstream NF-κB-mediated inflammatory cascades, while low dietary fiber limits SCFA-mediated counter-regulatory effects, creating a combined pro-inflammatory milieu [168,169].

In addition to excess saturated fats and refined carbohydrates, Western dietary patterns are also characterized by exposure to cholesterol oxidation products (oxysterols), which can arise during industrial food processing, prolonged storage, and high-temperature cooking of cholesterol-rich foods. These oxidized cholesterol derivatives possess strong pro-oxidant and pro-inflammatory biological activity and have increasingly been implicated in mechanisms linking Western dietary exposures with intestinal inflammation and epithelial barrier dysfunction [170,171,172].

Experimental studies indicate that oxysterols can induce oxidative stress and activate inflammatory signaling pathways such as NF-κB in intestinal epithelial cells, thereby contributing to disruption of epithelial barrier integrity and amplification of mucosal inflammatory responses. In parallel, Western-type diets rich in saturated fats have been shown to impair intestinal barrier function and increase epithelial permeability, further promoting inflammatory signaling in the gastrointestinal mucosa [149,173].

Recent integrative analyses further highlight how Western-style dietary patterns interact with gut barrier function, microbial metabolism, and host immune signaling to sustain chronic low-grade inflammation relevant to gastrointestinal and systemic disease [174].

Additives commonly present in ultra-processed foods are increasingly recognized as biologically active co-exposures rather than inert ingredients. Experimental models suggest that certain emulsifiers (e.g., carboxymethylcellulose, polysorbate-80) can disrupt mucus architecture, promote bacterial encroachment, and trigger low-grade inflammation at exposure levels below traditional toxicity thresholds; although human data remain limited, these findings support a precautionary perspective, particularly in susceptible populations [175].

### 4.2. Mediterranean Dietary Pattern (Bioactive Density, SCFA Support, and Inflammation Resolution)

The Mediterranean diet (MD) is defined by high intake of fruits/vegetables/legumes/nuts/whole grains, olive oil as the main fat, moderate fish/poultry, low red/processed meat, and (in many definitions) moderate wine with meals [175,176]. The PREDIMED trial established systemic benefits and catalyzed mechanistic work on inflammation and microbial remodeling [177]. A 2024 synthesis reported consistent microbiome benefits with MD adherence, including enrichment of taxa linked to SCFA production and barrier function (e.g., *Bifidobacterium*, *F. prausnitzii*, *Roseburia*, *A. muciniphila*) [178]. Short interventions (e.g., 8 weeks) have been associated with increased microbiome richness and reductions in systemic inflammatory markers such as C-reactive protein (CRP) in some cohorts [179].

The MD’s anti-inflammatory profile is best understood as a synergy of bioactive exposures rather than a single nutrient effect. Extra-virgin olive oil (EVOO) provides oleic acid (linked to peroxisome proliferator-activated receptor (PPAR)-γ signaling) and polyphenols (oleuropein, hydroxytyrosol, oleocanthal) that exhibit antioxidant effects and NF-κB modulation [104]. Polyphenols from plant foods and wine undergo microbial biotransformation into metabolites (e.g., urolithins, phenolic acids) that can influence immune signaling and epithelial function [180]. High fermentable fiber availability supports SCFA production and butyrate-mediated HDAC inhibition and Treg-related immune regulation. In parallel, ω-3 polyunsaturated fatty acids (eicosapentaenoic acid/docosahexaenoic acid) (PUFAs (EPA/DHA)) support the generation of specialized pro-resolving mediators (resolvins/protectins/maresins), shifting inflammatory biology toward active resolution rather than only suppressing initiation [181].

In addition to its nutrient composition, the Mediterranean dietary pattern provides a diverse array of substrates that interact with the gut microbiome and host metabolic signaling pathways. Microbiome-mediated metabolism of dietary components can generate a broad spectrum of bioactive metabolites capable of influencing host physiology, including pathways linking microbial metabolism with neuroendocrine and immune regulation along the gut–brain axis [182]. Experimental studies further demonstrate that commensal bacteria can modulate host metabolic functions through the production of signaling molecules, including microbiota-derived metabolites that influence intestinal and systemic physiology [183]. Moreover, the gut microbiota contributes to host micronutrient availability through microbial biosynthesis of vitamins and other bioactive compounds, highlighting the integrative relationship between diet composition, microbial metabolism, and host nutritional status [184].

These biological features position the Mediterranean dietary pattern as a microbiome-supportive and anti-inflammatory nutritional framework relevant to gastrointestinal health. The combination of fermentable fiber, polyphenol-rich plant foods, and unsaturated fatty acids promotes microbial diversity, short-chain fatty acid production, and modulation of inflammatory signaling pathways within the intestinal mucosa. Through these mechanisms, Mediterranean-style dietary patterns may contribute to improved epithelial barrier function, immune regulation, and metabolic homeostasis within the gut ecosystem.

### 4.3. Low-FODMAP Diet (Fermentation/Osmotic Load Reduction with Microbiome Trade-Offs)

The low-FODMAP diet restricts poorly absorbed, rapidly fermented short-chain carbohydrates (fructose in excess, lactose, fructans, galacto-oligosaccharides (GOS), and polyols) and was developed to reduce luminal fermentation and osmotic load within the intestine [185]. Network meta-analysis and meta-analytic syntheses support it as the most evidence-based dietary approach for IBS, with response rates commonly reported between 50–80% in trials, and significant improvements across pain, bloating, flatulence, and bowel habit outcomes [186,187].

Biologically, symptom benefits reflect reduced luminal water (osmotic effect) and reduced fermentation-related gas generation, which is particularly meaningful in the context of visceral hypersensitivity [188]. However, low-FODMAP restriction also reduces prebiotic substrate availability and consistently decreases *Bifidobacterium* abundance and sometimes overall SCFA output, raising concerns about long-term implementation without reintroduction/personalization [189,190]. Current best practice remains the 3-phase model (elimination → systematic reintroduction → individualized maintenance), optionally supported by targeted low-FODMAP prebiotic strategies to preserve microbiome function.

A key development is the emergence of microbiome predictors of response. IBS microbiome subtypes (e.g., a “pathogenic enrichment” subtype) may experience greater symptom improvement and shifts toward a healthier composition during low-FODMAP implementation, supporting a future precision nutrition pathway, pending larger validation cohorts [191].

### 4.4. Plant-Based Dietary Patterns (Fiber-Centric Microbiome Support with IBD Practicality Constraints)

Plant-based dietary patterns span vegan to flexitarian approaches but share high fiber intake and high polyphenol/antioxidant exposure, with reduced saturated fat and reduced exposure to heme iron/heterocyclic amines from red/processed meat [192]. Cross-sectional and intervention data associate plant-predominant patterns with increased microbial diversity and enrichment of fiber-degrading/SCFA-producing taxa (e.g., *Prevotella*, *Ruminococcus*, Lachnospiraceae members), often within days of dietary change and stabilizing over weeks [193,194].

The biological relevance of plant-based dietary patterns largely reflects their high content of fermentable carbohydrates and resistant starch, which serve as key substrates for microbial metabolism in the colon. Fermentation of these compounds supports the production of short-chain fatty acids, particularly butyrate, acetate, and propionate, metabolites that contribute to epithelial energy supply, mucosal barrier maintenance, and immune regulation within the intestinal environment [195]. Experimental and multi-omics studies further demonstrate that diets enriched in resistant starch can substantially influence microbial gene expression, metabolic pathways, and host–microbiome metabolic interactions, underscoring the capacity of plant-derived carbohydrates to shape the functional activity of the gut microbiota [196]. From a nutritional perspective, however, strict plant-based dietary patterns require careful planning to ensure adequate intake of nutrients that may be less abundant or less bioavailable in plant sources, including vitamin B12, iron, zinc, calcium, and long-chain omega-3 fatty acids. Recent nutritional analyses further highlight that the health effects of plant-forward diets depend not only on macronutrient composition but also on micronutrient adequacy and overall dietary quality, emphasizing the importance of balanced plant-based dietary design in maintaining metabolic and gastrointestinal health.

### 4.5. Gluten-Free Diet (Essential Therapy for Celiac Disease; Nuanced Role in NCGS/IBS Overlap)

The gluten-free dietary pattern is characterized by the exclusion of gluten-containing cereals, primarily wheat, rye, and barley, and their derivatives. In practice, this pattern relies on naturally gluten-free foods such as rice, maize, legumes, fruits, vegetables, and alternative grains including buckwheat or quinoa, as well as commercially produced gluten-free products. Because gluten-containing cereals represent major sources of dietary fiber and fermentable substrates in many populations, their exclusion can substantially modify macronutrient composition and fermentable carbohydrate availability within the diet [197,198].

From a biological perspective, the relevance of gluten-containing cereals in gastrointestinal physiology relates not only to gluten peptides themselves but also to other wheat components such as fructans (a class of FODMAPs) and amylase–trypsin inhibitors (ATIs), which may influence immune activation and intestinal signaling pathways. These components can interact with epithelial and innate immune mechanisms, contributing to mucosal immune responses and barrier modulation in susceptible individuals [199,200].

The gluten-free dietary pattern can also influence gut microbiome composition through changes in fermentable substrate availability and fiber intake. Reduced consumption of whole-grain cereals may decrease exposure to prebiotic fibers that support beneficial microbial taxa, potentially altering microbial diversity and metabolic output. Conversely, dietary patterns emphasizing naturally gluten-free whole foods may maintain microbial diversity and metabolic activity through alternative fiber sources and plant-derived substrates [201,202].

These distinguishing features, biological signatures, microbiome effects, and clinical applications of the principal dietary patterns discussed are compared in Table 4.

### 4.6. Integrative Perspective

Across patterns, two pragmatic principles emerge: Mediterranean/plant-predominant patterns function as broadly protective “base diets” for many GI conditions; targeted therapeutic diets (low-FODMAP, GFD) should be applied with defined indications and structured reintroduction/personalization to reduce unnecessary long-term restriction. The following section examines how these dietary patterns influence the pathophysiology and clinical course of specific gastrointestinal disorders.

## 5. Pathophysiological Impact of Dietary Patterns Across GI Conditions

GI disorders influenced by dietary exposures can be broadly grouped into several pathophysiological categories, including immune-mediated enteropathy (celiac disease), disorders of gut–brain interaction (irritable bowel syndrome), inflammatory bowel diseases (Crohn’s disease and ulcerative colitis), upper gastrointestinal inflammatory conditions (gastritis, *Helicobacter pylori* infection, and peptic ulcer disease), and carbohydrate malabsorption syndromes such as lactose intolerance. Across these categories, dietary factors influence disease processes through multiple biological mechanisms, including antigen exposure (e.g., gluten), fermentation and osmotic load (FODMAPs and lactose), microbiome–metabolite interactions (such as short-chain fatty acids and bile acids), modulation of epithelial barrier integrity, and activation of inflammatory signaling pathways. At the same time, gastrointestinal disorders themselves can modify dietary tolerance, nutrient bioavailability, digestive physiology, and microbiome composition, reinforcing the bidirectional nature of diet–disease interactions increasingly recognized in microbiome-focused research [39,40].

### 5.1. Celiac Disease: Gluten Elimination as Causal Therapy (Plus Monitoring and “Next-Generation Adjuncts”)

Celiac disease remains the clearest example of a diet-dependent immune disorder: gluten peptides trigger adaptive HLA-restricted responses and innate IL-15-driven epithelial injury, producing villous atrophy and malabsorption [203,204]. Despite strict dietary therapy, mucosal healing is heterogeneous; pooled analyses report 34% recovery at 2 years and 66% at 5 years, and a substantial minority experience persistent symptoms and/or mucosal inflammation often due to inadvertent exposure [205,206]. Serology can normalize before histology, and gluten immunogenic peptides in stool/urine are emerging adherence biomarkers with very high performance for detecting low-dose exposures that may sustain injury [207]. Nutritionally, the gluten-free diet (GFD) requires planned fiber and micronutrient replacement due to frequent deficits in iron, folate/B12, vitamin D, calcium, and zinc, and microbiome shifts (e.g., reduced *Bifidobacterium*) that may reflect reduced whole-grain/prebiotic intake unless actively addressed [208]. Adjunct therapies (enzymes, barrier modulators, tissue transglutaminase 2 (TG2) inhibitors such as ZED1227/TAK-227) are promising but not yet standard; thus, strict lifelong GFD remains foundational [209].

### 5.2. IBS: Symptom-Targeted Dietary Sequencing (Low-FODMAP as Escalation)

IBS reflects a multifactorial disorder involving gut–brain axis dysregulation, visceral hypersensitivity, altered motility, low-grade immune activation, and microbiome perturbations, providing a strong biological rationale for dietary intervention while also explaining the marked interindividual variability in response [210]. Among available strategies, the low-FODMAP diet has the most robust randomized and meta-analytic support; network meta-analyses and umbrella reviews consistently demonstrate clinically meaningful improvements in global symptoms as well as key domains such as abdominal pain and bloating [198,199]. However, because fermentable carbohydrate restriction can reduce microbial diversity and beneficial taxa, its use is recommended within a structured three-phase model (restriction, reintroduction, personalization) to minimize unnecessary long-term exclusion [211,212].

Emerging approaches are exploring microbiome-informed personalization strategies designed to retain dietary diversity while maintaining symptom control, though current evidence remains preliminary and requires cautious clinical translation [213]. Recent work further emphasizes that IBS dietary management should be individualized, integrating symptom phenotyping, microbiome considerations, and patient tolerance to optimize outcomes rather than relying on uniform restriction [214].

In practice, management typically begins with general dietary advice, progresses to a low-FODMAP intervention in non-responders, and then transitions toward personalized modification; wheat or gluten restriction may benefit selected patients, although fructans rather than gluten are often the principal symptom drivers [214,215].

### 5.3. Crohn’s Disease and UC: Mediterranean/Whole-Food Patterns as Foundation, Induction Diets for Specific Contexts

In Crohn’s disease, diet associations are consistent and mechanistically aligned with dysbiosis and barrier dysfunction. Mediterranean-style diets are increasingly supported as an adjunctive foundation: prospective data in newly diagnosed patients link adherence to improved inflammatory markers and reduced dysbiosis, with multi-omic shifts (e.g., bile acids and metabolites) supporting biological plausibility [216]. Emerging clinical and observational studies further indicate that adherence to Mediterranean-style dietary patterns may influence intestinal inflammation, microbiota composition, and metabolic parameters in patients with inflammatory bowel disease [217]. Dietary intervention in Crohn’s disease dietary inflammatory index for Crohn’s disease (DINE-CD) suggests symptom remission rates comparable to more restrictive protocols, with better long-term feasibility [218]. Pediatric and subclinical inflammation datasets (e.g., fecal calprotectin associations) reinforce that dietary quality may matter even under biologic therapy [219]. For induction, exclusive enteral nutrition remains first-line in pediatric disease due to strong remission and mucosal healing outcomes, while Crohn’s disease exclusion diet (CDED) offers a pragmatic whole-food approach excluding emulsifiers and specific additives, often with partial enteral nutrition support [220,221]. Clinically, fiber restriction should be individualized and reserved for strictures or active flare intolerance rather than applied universally.

In UC, colonic localization increases relevance of luminal metabolites (notably butyrate). Mediterranean-style interventions have demonstrated reductions in inflammation with microbiome shifts in randomized controlled trial (RCT) settings [222,223] with supportive observational signals in pouch-related contexts [224]. Practical UC guidance typically involves fiber modulation by phase: lower insoluble fiber during flares and active symptoms, but restoration during remission to support SCFA production and microbial diversity. Specific components (e.g., limiting red/processed meat and additive-heavy UPFs; supporting ω-3 intake) remain mechanistically plausible but variably supported in clinical trials [225,226].

### 5.4. Gastritis/H. pylori and Peptic Ulcer Disease: Diet as Adjunct, Not Replacement

For *H*. *pylori*-associated gastritis, eradication therapy remains primary, but dietary adjuncts (polyphenols such as extra virgin olive oil (EVOO) components, sulforaphane, cranberry-derived compounds) show in vitro activity and limited/modest human effects; they may be reasonable adjuncts but not substitutes, particularly in high resistance settings or after treatment failure [227,228,229,230]. In gastric health more broadly, salt and preserved foods associate with cancer risk and may exacerbate mucosal injury and *H. pylori* virulence, whereas fruit/vegetable intake and Mediterranean-like patterns are generally protective [231]. Peptic ulcer disease (PUD) management remains anchored to *H. pylori* eradication and NSAID strategy; dietary measures are supportive (symptom control, mucosal protection) with limited RCT-grade evidence for specific foods, though fiber and polyphenol-rich patterns are plausible supportive frameworks [232,233,234].

### 5.5. Lactose Intolerance: Threshold-Based Management with Nutrient Protection

Lactose intolerance is best managed via threshold identification rather than blanket avoidance. Symptoms depend on dose, residual lactase activity, transit time, microbiome fermentation profile, and visceral sensitivity; many tolerate 12–15 g lactose when consumed with meals [235,236]. Practical tools include choosing low-lactose foods (hard cheeses), yogurt with live cultures, lactose-free milk, lactase supplements, and gradual reintroduction for possible colonic adaptation (evidence variable) [237]. Because dairy restriction risks calcium/vitamin D inadequacy, counseling on fortified alternatives and dietary calcium sources is essential [238]. A comparative overview of the dietary strategies, underlying targets, and practical implementation considerations across major gastrointestinal conditions is presented in Table 5.

## 6. Probiotics, Prebiotics, Synbiotics, and Postbiotics: Biotic Adjuncts to Dietary Intervention

Because dietary patterns reshape microbiome structure and function, biotic strategies can be positioned as adjuncts that support microbial resilience during dietary therapy (e.g., low-FODMAP), target specific outcomes (e.g., UC remission maintenance), or deliver defined microbial functions (e.g., postbiotic butyrate delivery).

### 6.1. Definitions and Molecular Routes (The International Scientific Association for Probiotics and Prebiotics (ISAPP) Framework)

ISAPP defines probiotics as live microorganisms conferring benefit when administered in adequate amounts; prebiotics as selectively utilized substrates; synbiotics as combinations (synergistic or complementary); and postbiotics as preparations of inanimate microorganisms and/or components that confer benefit [239,240]. Clinically, the most important principle is specificity: probiotic effects are strain-specific, prebiotic effects are substrate- and microbiome-dependent, and postbiotic effects are component- and delivery-dependent.

Across categories, shared mechanistic routes include: barrier reinforcement (tight junction/mucin support), immune modulation (increasing IL-10 and regulatory responses, reducing TNF-α/IL-6), ecological effects (competitive exclusion, bacteriocin production), and functional metabolite support (especially SCFAs such as butyrate) [241,242].

### 6.2. Probiotics: Evidence Patterns by Condition (IBS, UC, and Crohn’s Disease)

IBS has the broadest probiotic evidence base but also substantial heterogeneity. Meta-analytic syntheses suggest modest benefits for global symptoms and pain, with variable certainty depending on strain; adverse events are generally not increased [90,243,244]. Practically, strain selection should be conservative and evidence-led; the literature most often highlights *Bifidobacterium* and *Lactobacillus* strains, with certain named strains used commonly in practice (e.g., *B. infantis* 35624, *L. plantarum* 299v, *B. coagulans* MTCC5260), while recognizing that replication and standardization remain limitations [245]. The most clinically useful positioning is: probiotics as a time-limited trial (e.g., 4–8 weeks) integrated with diet, not as standalone therapy.

In IBD, evidence diverges sharply between UC and Crohn’s disease. Updated syntheses support benefit in UC for remission induction/maintenance in some contexts, with VSL#3/Visbiome and *E. coli* Nissle 1917 often cited as higher-evidence options, and strong data for pouchitis prevention/treatment [246,247]. In Crohn’s disease, trials are largely negative for induction or maintenance, with only limited signals in select pediatric settings [248]. This UC–CD divergence is clinically important and should be stated explicitly to prevent overgeneralization.

### 6.3. Prebiotics and Synbiotics: Function Depends on Fermentation “Fit”

Prebiotic substrates, including inulin, fructo-oligosaccharides (FOS), galacto-oligosaccharides (GOS), lactulose, as well as resistant starch and polydextrose, are best conceptualized as modulatory tools aimed at enhancing short-chain fatty acid (SCFA) production and supporting the growth of beneficial microbial taxa. However, their physiological impact is highly context-dependent, varying according to baseline microbiome composition, metabolic functionality, and dose tolerability [192,249,250,251]. Emerging clinical and translational evidence further indicates that even structurally related fructan prebiotics are not interchangeable: differences in polymer chain length and fermentability (e.g., longer-chain inulin versus shorter-chain FOS) can lead to distinct metabolic and functional outcomes, including divergent effects on host metabolic endpoints and microbial biosynthetic pathways such as folate metabolism [192]. These findings reinforce the view that the term “prebiotic” does not represent a single, uniform exposure but rather a heterogeneous class of substrates with differential ecological and metabolic effects that must be matched to host–microbiome characteristics [250].

In GI disorders, symptomatic limits often constrain dosing, and clinical outcome data remain mixed; network analyses do not consistently show prebiotics outperforming probiotics in IBS [252,253].

Synbiotics aim to pair organisms with their preferred substrates and may add value where either component alone is insufficient. Some trials (including combinations incorporating microencapsulated butyrate + probiotics + scFOS) suggest symptom improvement in IBS, but optimization of pairing, ratios, and delivery remains a research frontier [254,255,256].

### 6.4. Postbiotics: Stability and Safety Advantages, Delivery Challenges

Postbiotics are appealing due to stability and safety (no live translocation risk), but they require effective delivery of the active component. Butyrate is the prototypical postbiotic with HDAC inhibition, GPCR signaling, barrier support, NF-κB modulation, and Treg promotion; oral delivery requires encapsulation/esterification strategies to reach the colon [257,258,259,260]. Cell wall components (MDP, LTA) can modulate innate immunity through NOD2/TLR-related pathways and show experimental anti-inflammatory or metabolic effects, though translation to routine GI practice remains early [261,262]. The principal categories of biotic interventions, their definitions, clinical contexts, and practical considerations are summarized in Table 6.

### 6.5. Practical Integration with Dietary Therapy and Safety

Biotics should generally be positioned as adjuncts: for example, low-FODMAP diet can reduce *Bifidobacterium*, supporting a rationale for targeted probiotic co-administration and structured reintroduction of prebiotic foods [100]. In UC, evidence-based probiotic formulations may be combined with 5-ASA in remission maintenance. In celiac disease, microbiome shifts on GFD provide a rationale for emphasizing naturally gluten-free prebiotic-rich foods and considering probiotics selectively, while always prioritizing strict gluten exclusion.

Safety is favorable in immunocompetent individuals, but caution is warranted in severely immunocompromised patients, those with central lines, and other high-risk contexts; postbiotics may offer theoretical advantages where viability-related risk is unacceptable [263].

## 7. Limitations of Current Evidence and the Emerging Paradigm of Precision Nutrition

Although the preceding sections demonstrate consistent associations between dietary patterns and gastrointestinal health, important methodological and conceptual limitations constrain causal inference and clinical translation. Dietary interventions differ fundamentally from pharmacological trials: diets are complex, multi-component exposures embedded within food matrices, precluding true placebo control and blinding. Adherence is difficult to verify objectively and often relies on self-report, while effect sizes tend to be modest and highly context dependent. These features contribute to substantial heterogeneity across studies and limit the proportion of nutrition recommendations supported by high-level randomized evidence.

In gastroenterology, these challenges are amplified by disease-specific factors. In irritable bowel syndrome, inconsistent stratification by subtype and heterogeneous outcome measures limit comparability across trials. In IBD, dietary effects are difficult to disentangle from relapsing disease courses and concomitant pharmacotherapy. In CD, reliance on self-reported gluten avoidance frequently overestimates true adherence, while objective biomarkers remain underused. Control group selection further complicates interpretation, as habitual diets introduce lifestyle confounding and structured comparator diets may exert unintended biological effects.

Beyond these methodological constraints, the most fundamental limitation of population-based dietary guidance is the marked interindividual variability in response to identical foods. Large-scale metabolic studies demonstrate wide variation in postprandial glycemic and lipemic responses, even among genetically identical individuals, with genetic factors explaining only a modest proportion of this variability. Gut microbiome composition consistently emerges as a key determinant of individual responses, often accounting for more variability than macronutrient composition alone. These findings challenge the conceptual foundation of one-size-fits-all dietary recommendations.

The convergence of these limitations has driven the emergence of precision nutrition, an approach that seeks to individualize dietary advice using integrated biological data rather than population averages. Recent digital-health frameworks further illustrate how individualized nutrition strategies can be operationalized in clinical practice by integrating dietary intake data, metabolic biomarkers, and longitudinal patient monitoring to guide personalized dietary recommendations and follow-up [264]. Contemporary scholarship describes precision nutrition as a paradigm shift enabled by advances in multi-omics profiling, digital health technologies, and machine-learning analytics, which together allow the integration of genetic, metabolic, microbiome, and lifestyle data to generate individualized dietary recommendations and predict heterogeneous responses to foods and interventions [265].

**Figure 3:**
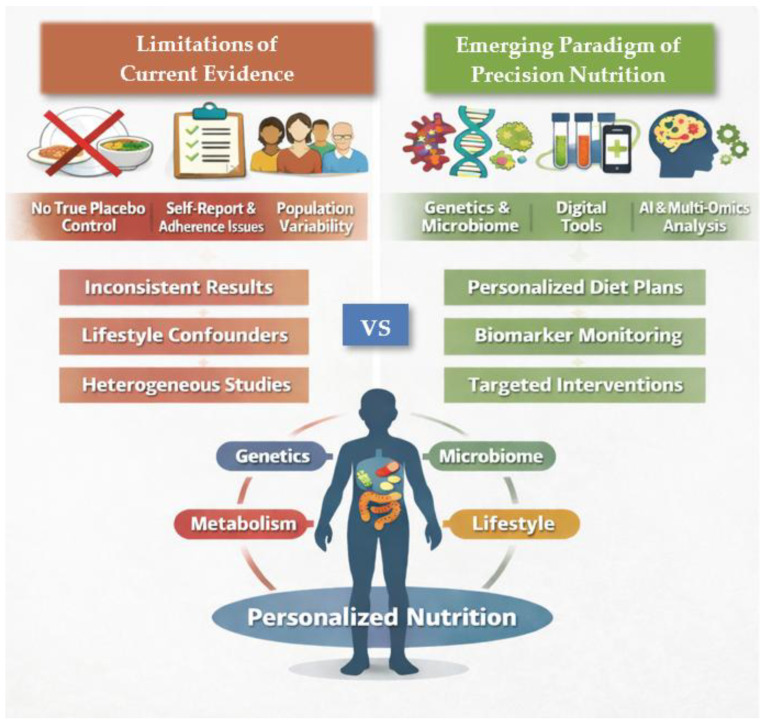
Limitations of current dietary evidence and the emergence of precision nutrition in gastroenterology. Traditional dietary studies are affected by methodological constraints such as the absence of true placebo controls, reliance on self-reported adherence, and heterogeneous study designs. These limitations contribute to inconsistent results and are further complicated by substantial interindividual variability in responses to identical foods. Precision nutrition aims to address this variability by integrating genetic, microbiome, metabolic, and lifestyle data to generate individualized dietary recommendations. The figure was created in Biorender, by Muntean Calin. (2026) https://BioRender.com/ydud3pe (last accessed on 25 February 2026) and PowerPoint.

Microbiome-informed algorithms have demonstrated superior predictive performance for postprandial metabolic responses compared with traditional nutrient-based approaches, and early gastroenterology trials suggest that personalized dietary strategies may achieve symptom control comparable to standardized therapeutic diets while preserving microbiome diversity. The interacting sources of methodological bias, biological variability, and emerging precision-nutrition solutions are schematically summarized in Figure 3.

Despite its promise, precision nutrition remains constrained by technical and practical barriers. Current models explain only a limited proportion of response variability, microbiome composition is temporally dynamic, and dietary intake assessment remains imperfect even with digital tools. Implementation requires infrastructure rarely available in routine care, raising concerns regarding cost, equity, data privacy, and algorithmic bias. Consequently, precision nutrition is unlikely to replace conventional dietary guidance in the near term.

Until this paradigm matures, clinical practice must integrate existing evidence pragmatically. Dietary patterns, rather than isolated nutrients, should remain the foundation of recommendations, with Mediterranean and plant-forward approaches offering the most consistent benefit across gastrointestinal conditions. Restrictive therapeutic diets should be implemented with professional supervision, structured reintroduction, and nutritional monitoring. Precision nutrition is likely to enter practice incrementally, particularly for conditions characterized by high response variability, serving as a complementary rather than disruptive evolution of dietary therapy.

## 8. Materials and Methods

This work was conducted as a narrative review integrating epidemiological, mechanistic, and clinical evidence on the role of dietary patterns in gastrointestinal (GI) health and disease. Although the review was not designed as a formal systematic review, a structured literature search and screening approach was applied to improve transparency and reproducibility. A narrative framework was deliberately chosen to allow conceptual synthesis across heterogeneous study designs, including prospective cohort studies, randomized controlled trials, experimental and translational mechanistic investigations, and microbiome-focused research. Given the complexity of dietary exposures and the multifactorial pathophysiology of gastrointestinal disorders, a purely systematic or meta-analytic framework was considered insufficient to capture the breadth of biological mechanisms, disease-specific nuances, and clinical contexts addressed in this review.

A comprehensive literature search was conducted using PubMed/MEDLINE, Scopus, and the Web of Science Core Collection. The search strategy combined controlled vocabulary terms and free-text keywords related to dietary patterns (including Mediterranean, Western, low-FODMAP, plant-based, and gluten-free diets), gastrointestinal disorders (celiac disease, irritable bowel syndrome, Crohn’s disease, ulcerative colitis, gastritis, peptic ulcer disease, and lactose intolerance), and mechanistic pathways linking diet to gastrointestinal physiology and pathology (gut microbiota, short-chain fatty acids, epithelial barrier function, immune signaling, epigenetic regulation, and precision nutrition). Searches focused primarily on articles published between January 2015 and May 2025, reflecting a period of rapid advances in microbiome science, nutritional epidemiology, and systems-based dietary research. Reference lists of key reviews, meta-analyses, and clinical guidelines were manually screened to identify additional relevant publications.

The literature search initially identified 1486 records across the three electronic databases. After removal of 312 duplicate records, 1174 articles remained for title and abstract screening. During this stage, 938 records were excluded because they did not address dietary patterns in relation to gastrointestinal disorders, focused on unrelated disease areas, or lacked sufficient methodological relevance to the objectives of this review.

The full texts of 236 articles were subsequently assessed for eligibility. Of these, 88 articles were excluded for the following reasons: lack of direct evaluation of diet–gastrointestinal relationships (n = 39), insufficient methodological description or unclear study design (n = 18), publication type such as editorials or case reports (n = 21), or language other than English (n = 10).

**Figure 4:**
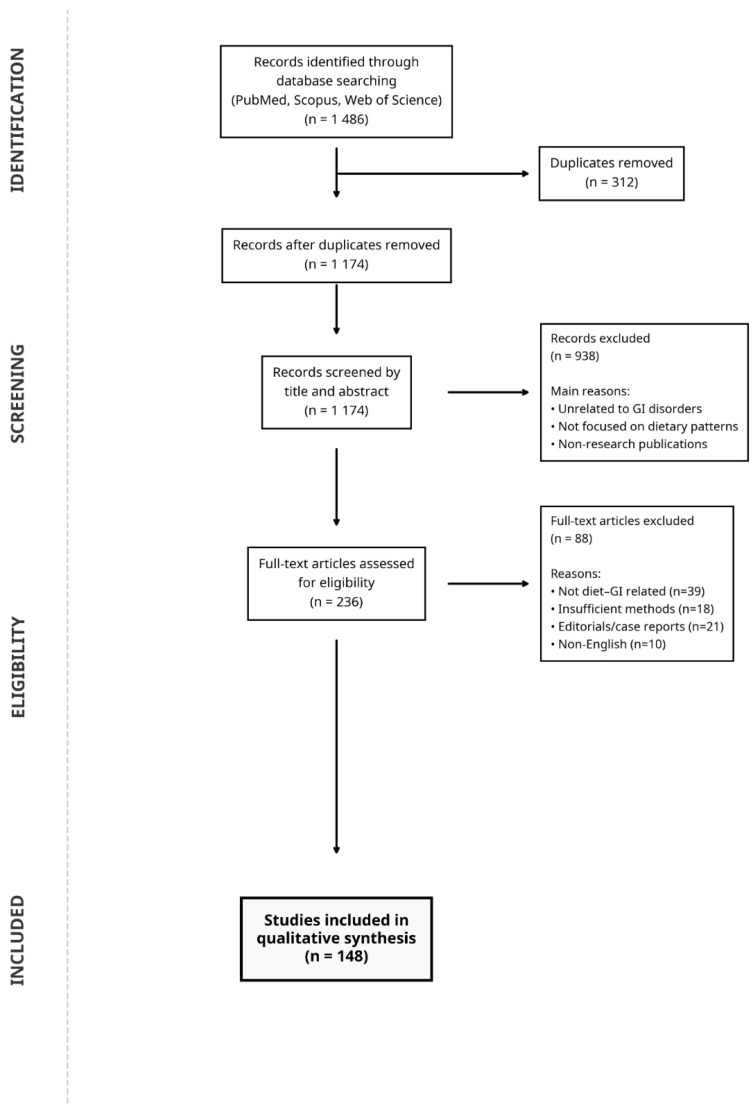
Flow diagram of the literature search and study selection process.

Ultimately, 148 publications met the eligibility criteria and were included in the qualitative synthesis. Additional references cited throughout the manuscript were included to provide mechanistic background, clinical context, and supporting epidemiological evidence beyond the studies captured through the structured screening process. The literature selection process is summarized in Figure 4.

Eligible studies included human observational studies, randomized controlled trials, systematic reviews and meta-analyses with clearly described methodologies, as well as experimental and translational studies providing insight into diet–microbiome–immune interactions relevant to gastrointestinal health. Articles were limited to those published in English. Case reports, editorials, and studies lacking sufficient methodological detail were excluded unless they provided unique mechanistic perspectives directly informing the conceptual framework of the review.

Data extraction focused on study design, population characteristics, dietary exposure definitions and assessment methods, gastrointestinal outcomes and diagnostic criteria, and reported molecular endpoints, including microbiome composition, microbial metabolites, inflammatory signaling pathways, and epigenetic markers. Where applicable, effect estimates such as relative risks, odds ratios, hazard ratios, and corresponding confidence intervals were considered. Rather than quantitative pooling, evidence was synthesized using a thematic and biological integration strategy, grouping findings according to dietary pattern, biological pathway, and disease phenotype. Priority was given to consistency across independent cohorts, convergence between mechanistic and clinical data, and relevance to clinical decision-making.

The overall strength of evidence supporting dietary interventions across gastrointestinal conditions was assessed qualitatively, taking into account study design hierarchy, sample size, duration of follow-up, risk of bias, and consistency of findings across populations. The appraisal reflects consideration of the totality of available evidence for each condition rather than reliance on individual studies. Particular attention was paid to potential confounders relevant to gastroenterology, including concomitant pharmacological therapies, disease activity status, and baseline nutritional status.

## 9. Conclusions and Future Directions

This narrative review integrates epidemiological trends, mechanistic evidence, and clinical studies to clarify the role of dietary patterns in gastrointestinal health. Collectively, the data support the view that diet is a biologically active determinant of gastrointestinal physiology and disease, acting through interconnected mechanisms involving the gut microbiome, immune regulation, epithelial barrier function, oxidative balance, and epigenetic modulation. These pathways link dietary exposures to both short-term functional responses and long-term disease trajectories.

Across GI conditions, dietary patterns, rather than isolated nutrients, emerge as the most clinically meaningful unit of intervention. In this context, the Mediterranean dietary pattern consistently represents a sustainable anti-inflammatory framework, associated with favorable microbiome profiles and improved outcomes across inflammatory bowel disease, functional gastrointestinal disorders, and gastric pathology. Conversely, Western dietary patterns characterized by high intake of ultra-processed foods and low fiber are repeatedly linked to dysbiosis, barrier dysfunction, and heightened inflammatory risk. To translate these concepts into practical guidance, Table 7 summarizes the overall strength of evidence and corresponding clinical recommendations across major GI conditions.

Strict gluten avoidance remains indispensable for celiac disease, while the low-FODMAP diet provides validated symptom control in irritable bowel syndrome when applied with structured reintroduction to preserve microbiome health. In Crohn’s disease, exclusive enteral nutrition and structured exclusion diets are effective for remission induction, whereas long-term management favors whole-food, minimally processed dietary patterns. Adjunctive biotic interventions may enhance dietary strategies when applied selectively and supported by strain-specific evidence, particularly in ulcerative colitis, pouchitis, and irritable bowel syndrome, while postbiotic approaches represent a promising translational extension of mechanistic insights.

From a clinical perspective, these findings underscore the need to integrate dietary assessment and counseling into routine gastroenterological care with rigor comparable to pharmacological management. Nutritional adequacy must be actively monitored whenever dietary restriction is imposed, and collaboration with specialized gastrointestinal dietitians should be considered standard practice for complex dietary interventions. Probiotic use should remain targeted, indication-specific, and time-limited, rather than empirical.

Looking forward, future research should prioritize standardized outcome measures, longer-duration intervention studies, and direct comparisons between dietary patterns. Mechanistic investigations aimed at identifying microbiome-derived mediators of dietary effects will support the development of targeted postbiotic therapies, while advances in nutrigenetics and systems biology may enable more precise patient stratification. Precision nutrition offers a compelling trajectory toward individualized dietary care, particularly for conditions characterized by marked interindividual variability, but its implementation must address issues of feasibility, equity, and interpretability.

In conclusion, diet has evolved from ancillary lifestyle advice to a mechanistically grounded therapeutic component of gastroenterology. Treating dietary intervention with the same scientific rigor as pharmacotherapy, while recognizing its cultural, social, and personal dimensions, represents both an evidence-based obligation and an opportunity to improve long-term gastrointestinal health.

## Figures and Tables

**Table 1:** Global epidemiology and diet links across major GI conditions (2015–2025).

Condition	Key Prevalence/ Incidence Metrics	Sex Pattern	Temporal Trend	Core Diet-Linked Associations (Direction)	Key Modifiers/Notes	Refs.
Crohn’s disease	High in North America/ Western Europe; rising in newly industrialized regions	slight female excess	Stabilizing incidence (West); increasing elsewhere	↑ inflammatory patterns; ↑ ultra-processed foods; ↓ fiber; ↑ Mediterranean adherence protective	Latency with westernization (15–30 yrs); confounding by meds possible	[41,42,43,44,45,50]
Ulcerative colitis	High in Western regions; increasing in Asia/other regions	no strong sex bias	Stabilizing (West); increasing elsewhere	Associations more heterogeneous than CD	Phenotype/ measurement heterogeneity	[46,47,48,49,50]
IBS (Rome III vs. IV)	Rome III 9% vs. Rome IV 4% (varies widely)	Female > male	“Stable” but criteria-dependent	Symptoms triggered by foods in many; low-FODMAP most evidence-based	Strong psych comorbidity influence; subtype variation	[55,56,57,58,59,60,61]
Celiac disease	Serology 1.4%; biopsy 0.7%	Female > male	Increasing incidence in many datasets	Gluten causal in susceptible HLA	First-degree relatives high prevalence	[62,63,64,65]
Peptic ulcer disease	Falling global AS incidence/ mortality	Male slightly > female	Declining overall; disparities persist	Salt/preserved foods, alcohol/smoking linked; diet may influence mucosal defense/*H. pylori*	*H. pylori*/NSAID key causes; access disparities	[66,67,68]
Chronic gastritis → cancer pathway	Very high global prevalence (parallels *H. pylori*)	variable	Persistent burden	↑ salt/preserved/smoked; ↓ fruit/veg; Mediterranean protective	Correa cascade context	[69,70,71,72]
NCGS (Non-celiac gluten sensitivity)	Wide estimates (method-dependent)	variable	unclear	Wheat/gluten exposure symptom-linked; may reflect fructans/ATIs in some	True prevalence lower under DBPC challenge	[73,74,75,76]
Lactose intolerance	68% globally (ethnicity-dependent)	no strong bias	stable	Dairy dose-dependent symptoms	Coexists with IBS; restriction response heterogeneous	[77,78,79,80]

AS, age-standardized; ATIs, amylase–trypsin inhibitors; CD, Crohn’s disease; DBPC, double-blind placebo-controlled; GI, gastrointestinal; *H. pylori*, *Helicobacter pylori*; HLA, human leukocyte antigen; IBS, irritable bowel syndrome; NCGS, non-celiac gluten sensitivity; NSAID, nonsteroidal anti-inflammatory drug; Rome III/IV, diagnostic criteria sets for functional gastrointestinal disorders developed by the Rome Foundation; ↑, increase or positive association; ↓, decrease or inverse association; >, higher prevalence in the specified group. → can lead to.

**Table 2:** Key signaling pathways modulated by diet in GI inflammation.

Pathway	Major Diet-Linked Activators	Major Diet-Linked Inhibitors/Modulators	Key Outputs Relevant to GI Disease	Notes	Refs.
TLR4–MyD88	LPS; SFA (palmitate/laurate)	Fiber-mediated ↓ endotoxemia; microbiome restoration	NF-κB activation; TNF-α/IL-1β/IL-6 induction	Central “gateway” for Western diet inflammation	[120,121,122,123,124]
NF-κB	Cytokines; TLR ligands; ROS	Butyrate (HDAC); ω-3; polyphenols; PPAR-γ	Cytokines/chemokines; COX-2/iNOS; barrier disruption	Integrator of immune + diet + microbe cues	[107,108,109,117,118,119,125,126,127,128,129]
MAPK (p38/JNK/ERK)	ROS; oxidative stress; H_2_O_2_	Antioxidant-rich patterns; SCFA	Cytokine induction; mRNA stabilization	Cross-talks with NF-κB via TAK1	[122,123,124]
PPAR-γ	—	Oleic acid; ω-3 PUFA; polyphenols; TZDs	Transrepression of NF-κB/AP-1/STAT	Reduced expression in IBD mucosa reported	[125,126,127,128,129]
NLRP3 inflammasome	Metabolic stress; mitochondrial ROS	Context-dependent (SCFA may induce protective IL-18)	IL-1β/IL-18 maturation	Distinguish pathologic vs. physiologic activation	[130,131]

5-ASA, 5-aminosalicylic acid; ATI, amylase–trypsin inhibitors; CDAI, Crohn’s Disease Activity Index; CDED, Crohn’s Disease Exclusion Diet; CRP, C-reactive protein; EEN, exclusive enteral nutrition; EVOO, extra-virgin olive oil; FODMAP, fermentable oligosaccharides, disaccharides, monosaccharides, and polyols; GFD, gluten-free diet; GIP, gluten immunogenic peptides; HDAC, histone deacetylase; IBD, inflammatory bowel disease; IBS-SSS, Irritable Bowel Syndrome Severity Scoring System; LTA, lipoteichoic acid; MDP, muramyl dipeptide; NF-κB, nuclear factor kappa-light-chain-enhancer of activated B cells; NOD2, nucleotide-binding oligomerization domain-containing protein 2; PPAR-γ, peroxisome proliferator-activated receptor gamma; RCT, randomized controlled trial; SCFA, short-chain fatty acids; TLR, Toll-like receptor; UPF, ultra-processed foods. ↓ decrease.

**Table 3:** Diet → microbiome metabolites → epigenetic/immune effects relevant to GI inflammation.

Dietary Exposure (Pattern/Component)	Dominant Microbial/Metabolic Shift	Key Metabolites	Primary Host Receptors/Epigenetic Enzymes	Downstream Functional Effects	Representative Refs.
Fermentable fiber (Mediterranean/plant-rich)	↑ SCFA-producers (Faecalibacterium, Roseburia, Bifidobacterium)	Butyrate, propionate, acetate	HDAC inhibition; GPR41/43/109A	↑ barrier integrity; ↑ Treg; ↓ NF-κB transcriptional tone	[23,24,25,26,27,29,30,105,106,107,108,109,110,111,112,113,114,115,116]
Western diet (high SFA/refined carbs, low fiber)	Dysbiosis favoring proteolytic/sulfate-reducers; ↑ endotoxemia	LPS, ROS-related byproducts; H_2_S	TLR4/MyD88; redox-sensitive kinases	↑ NF-κB/MAPK; ↑ cytokines; ↓ tight junctions	[11,12,120,121,122,123,124,132,133,134]
Polyphenol-rich foods	Microbial biotransformation; variable responder phenotypes	Phenolic metabolites	DNMT modulation; HAT changes; miRNAs	Context-dependent anti-inflammatory signaling; barrier support	[140,141]
ω-3 PUFA/olive oil (Mediterranean)	anti-inflammatory lipid mediators; microbiome shifts	lipid mediators	PPAR-γ activation; NF-κB transrepression	↓ inflammatory gene expression; immune modulation	[125,126,127,128,129]

DNMT, DNA methyltransferase; GPR41/43/109A, G protein-coupled receptor 41/43/109A; H_2_S, hydrogen sulfide; HAT, histone acetyltransferase; HDAC, histone deacetylase; LPS, lipopolysaccharide; MAPK, mitogen-activated protein kinase; miRNAs, microRNAs; MyD88, myeloid differentiation primary response 88; NF-κB, nuclear factor kappa-light-chain-enhancer of activated B cells; PPAR-γ, peroxisome proliferator-activated receptor gamma; PUFA, polyunsaturated fatty acids; ROS, reactive oxygen species; SCFA, short-chain fatty acids; SFA, saturated fatty acids; TLR4, Toll-like receptor 4; Treg, regulatory T cells. ↑, increase or positive association; ↓, decrease or inverse association.

**Table 4:** Comparative profile of five dietary patterns relevant to GI health.

Dietary Pattern	Defining Components	Key Mechanistic “Signature”	Typical Microbiome Direction	Best-Supported GI Use	Main Risks/Limitations	Refs.
Western	UPFs, refined carbs, SFA/trans fats, red/processed meat, sugar, sodium; low fiber	↑ permeability + endotoxemia; ↑ TLR4/MyD88 → NF-κB/MAPK; ↓ SCFA counter-regulation; additive/emulsifier effects	↓ diversity; ↓ *F. prausnitzii*, ↓ *Akkermansia*; ↑ Enterobacteriaceae, sulfate-reducers	Risk pattern for IBD and symptom aggravation	Chronic inflammation, mucus disruption, additive exposure	[150,151,152,153,154,155,156,157,158,159,160]
Mediterranean	plant foods, EVOO, fish, whole grains/legumes, polyphenols, ω-3; low red/processed meat	↑ PPAR-γ; ↓ NF-κB tone; ↑ SCFA/HDAC inhibition; ↑ resolution mediators (SPMs)	↑ diversity; ↑ SCFA producers; ↑ *Akkermansia*	IBD prevention/adjunct management; overall GI health	Adherence/cost/ cultural adaptation	[161,162,163,164,165,166,167,168,169,170,171]
Low-FODMAP	restriction of fermentable carbs; 3-phase model	↓ luminal water + gas; ↓ fermentation load; ↓ visceral triggering	↓ *Bifidobacterium* (often reversible); ↓ SCFA if prolonged strictness	IBS (best evidence); functional symptoms	Not a “forever” diet; requires reintroduction	[172,173,174,175,176,177,178]
Plant-based	high fiber/polyphenols; low SFA; reduced heme iron	↑ SCFA; ↓ pro-inflammatory exposures; antioxidant support	↑ diversity; ↑ fiber degraders/SCFA producers	IBD prevention/remission support (selected patients)	B12/iron/zinc/ ω-3 needs; flare/stricture tailoring	[179,180,181,182,183]
Gluten-free	eliminates wheat/rye/barley	celiac: removes causal trigger; NCGS: reduces wheat triggers (often fructans/ATIs)	can ↓ beneficial taxa if fiber drops	essential for celiac; subset NCGS/IBS	Nutritional quality varies; processed GFD products	[184,185,186,187,188,189]

ATIs, amylase–trypsin inhibitors; EVOO, extra virgin olive oil; FODMAP, fermentable oligosaccharides, disaccharides, monosaccharides, and polyols; GFD, gluten-free diet; GI, gastrointestinal; HDAC, histone deacetylase; IBD, inflammatory bowel disease; MAPK, mitogen-activated protein kinase; NCGS, non-celiac gluten sensitivity; NF-κB, nuclear factor kappa-light-chain-enhancer of activated B cells; PPAR-γ, peroxisome proliferator-activated receptor gamma; SCFA, short-chain fatty acids; SFA, saturated fatty acids; SPMs, specialized pro-resolving mediators; TLR4, Toll-like receptor 4; UPFs, ultra-processed foods; ω-3, omega-3 polyunsaturated fatty acids; ↑, increase or upregulation; ↓, decrease or downregulation. → can lead to.

**Table 5:** Dietary evidence across seven GI conditions (what works, why, and how to implement).

Condition	Diet Pattern(S) with Best Support	Primary Target Mechanism(S)	Evidence Signal (as You Wrote)	Practical Implementation Notes	Key Caveats	Refs.
Celiac disease	Strict GFD (essential)	Removes causal antigen; reduces adaptive/innate activation	Mucosal healing 66% at 5y; heterogeneity	Emphasize naturally GF whole grains + fiber; monitor micronutrients	Persistent symptoms from contamination; serology ≠ histology	[190,191,192,193,194,195,196]
IBS	Low-FODMAP (step-up); tailored reintroduction	↓ osmotic load + fermentation gas; ↓ visceral triggering	RR non-improvement 0.45; 50–80% response	3-phase protocol with dietitian; consider microbiome-support strategies	↓ *Bifidobacterium* if prolonged restriction	[197,198,199,200,201]
Crohn’s disease	Mediterranean as foundation; EEN/CDED for induction	↑ SCFA/anti-inflammatory signaling; ↓ additives/UPFs; whole-food remodeling	46% symptom remission at 6w (DINE-CD); favorable markers	Induction vs. maintenance strategies differ; tailor fiber in strictures	Adjunct to pharmacotherapy; adherence and phenotype matter	[202,203,204,205,206,207]
Ulcerative colitis	Mediterranean + remission-phase fiber optimization	↑ butyrate support; ↓ dysbiosis; reduced UPFs/additives	RCT: inflammation improvement + microbiome shifts	Flare: reduce insoluble fiber; remission: restore fermentable substrates	Evidence variable; avoid over-restriction	[208,209,210,211,212]
*H. pylori* gastritis	Standard eradication + diet adjuncts	Polyphenol antimicrobial; mucosal defense	Modest human effects; stronger in vitro	Consider EVOO/polyphenol-rich pattern as adjunct	Never replace eradication therapy	[213,214,215,216,217]
Peptic ulcer	*H. pylori*/NSAID strategy + supportive pattern	Mucosal protection; symptom minimization	Limited trial evidence for specific foods	Fiber/polyphenol-rich pattern; avoid irritants if symptomatic	“Bland diet” not evidence-based	[218,219,220]
Lactose intolerance	Threshold-based + alternatives	Reduce malabsorbed lactose load	Most tolerate 12–15 g with meals	Use yogurt/low lactose cheese/lactose-free; protect Ca/Vit D	Avoid unnecessary restriction; overlap with IBS	[221,222,223,224]

Ca, calcium; CDED, Crohn’s Disease Exclusion Diet; EEN, exclusive enteral nutrition; EVOO, extra virgin olive oil; GFD, gluten-free diet; GI, gastrointestinal; *H. pylori*, *Helicobacter pylori*; IBS, irritable bowel syndrome; RCT, randomized controlled trial; RR, relative risk; SCFA, short-chain fatty acids; UPFs, ultra-processed foods; Vit D, vitamin D; ↑, increase or beneficial upregulation; ↓, decrease or reduction in the specified factor; ≠, not equivalent.

**Table 6:** Biotic interventions in GI disorders: what to use, for whom, and why.

Category	ISAPP Definition	Typical Examples (As in Your Draft)	Best-Supported GI Indications	Mechanistic “Hook”	Practical Cautions	Refs.
Probiotics	Live microbes conferring benefit	*B. infantis* 35624; *L. plantarum* 299v; VSL#3/Visbiome; *E. coli* Nissle 1917	IBS (symptoms); UC (selected contexts); pouchitis	Barrier support, cytokine modulation, competitive exclusion, SCFA shifts	Strain-specific; quality/viability; time-limited trials	[225,226,227,228,229,230,231,232,233,234,235]
Prebiotics	Selectively utilized substrates	Inulin, FOS, GOS, lactulose, resistant starch	Function support (SCFA), adjunct in some contexts	SCFA production; bifidogenic effects	Dose-dependent bloating; responder variability	[236,237,238,239,240,241]
Synbiotics	Probiotic + prebiotic	Paired bifidobacteria + FOS/GOS; multi-strain + scFOS ± butyrate	IBS (emerging)	Survival/function support + substrate	Formulation complexity; optimal pairing unclear	[242,243,244]
Postbiotics	Inanimate microbes/components	Butyrate (encapsulated/tributyrin); MDP; LTA; heat-killed strains	Early clinical evidence; strong mechanistic rationale	Defined receptor /epigenetic signaling	Delivery matters; limited clinical validation	[90,245,246,247,248,249]

FOS, fructooligosaccharides; GI, gastrointestinal; GOS, galactooligosaccharides; IBS, irritable bowel syndrome; ISAPP, International Scientific Association for Probiotics and Prebiotics; LTA, lipoteichoic acid; MDP, muramyl dipeptide; SCFA, short-chain fatty acids; scFOS, short-chain fructooligosaccharides; UC, ulcerative colitis; ±, with or without the specified component.

**Table 7:** Summary of Evidence Grades and Clinical Recommendations by Gastrointestinal Condition.

Condition	Primary Dietary Intervention	Evidence Grade	Biotic Adjunct (If Applicable)	Key Monitoring Parameters	Refs.
Celiac disease	Strict gluten-free diet (lifelong, essential)	A (essential)	Probiotics for microbiome support (limited evidence)	Anti-tTG/DGP antibodies; gluten immunogenic peptides (GIP); follow-up biopsy at 1–2 years	[252,253,254,255,256,261,262,263]
Irritable bowel syndrome (IBS)	Low-FODMAP diet (3-phase) or Mediterranean diet	B (moderate)	Strain-specific probiotics (*B. infantis* 35624, *L. plantarum* 299v)	IBS-SSS; stool consistency; quality of life; optional microbiome profiling	[254,257,258,259,265]
Crohn’s disease	Mediterranean diet (maintenance); EEN or CDED (induction)	B (moderate)	Probiotics not recommended (no consistent efficacy)	CDAI; fecal calprotectin; CRP; nutritional status; fiber tolerance in strictures	[255,257,261,264]
Ulcerative colitis	Mediterranean diet; phase-adapted fiber optimization	B–C	VSL#3/Visbiome + 5-ASA (remission); *E. coli* Nissle 1917	Mayo score; fecal calprotectin; endoscopy when indicated	[255,257,258,259,265]
*Helicobacter pylori* gastritis	Standard eradication therapy; Mediterranean diet as adjunct	C (adjunctive)	Selected probiotics may support eradication (strain-specific)	Urea breath test post-eradication; salt intake reduction	[261,265]
Lactose intolerance	Individual lactose threshold identification; lactose-reduced options	B (moderate)	β-galactosidase-producing probiotics (variable evidence)	Calcium and vitamin D adequacy; bone health assessment	[258,260,261]

5-ASA, 5-aminosalicylic acid; anti-tTG/DGP, anti-tissue transglutaminase/deamidated gliadin peptide antibodies; *B. infantis*, *Bifidobacterium infantis*; CDAI, Crohn’s Disease Activity Index; CDED, Crohn’s Disease Exclusion Diet; CRP, C-reactive protein; EEN, exclusive enteral nutrition; E. coli, *Escherichia coli*; FODMAP, fermentable oligosaccharides, disaccharides, monosaccharides, and polyols; GI, gastrointestinal; GIP, gluten immunogenic peptides; IBS, irritable bowel syndrome; IBS-SSS, IBS Severity Scoring System; *L. plantarum*, *Lactiplantibacillus plantarum*; VSL#3/Visbiome, multi-strain probiotic formulations; β-galactosidase, lactase enzyme. Evidence grades: A = multiple high-quality RCTs with consistent results; B = limited RCTs or consistent observational data; C = primarily observational or mechanistic evidence.

## Data Availability

No new data were created or analyzed in this study. Data sharing is not applicable to this article.

## Abbreviations

The following abbreviations are used in this manuscript:

5-ASA	5-Aminosalicylic acid
AI	Artificial intelligence
APC	Annual percentage change
ASIR	Age-standardized incidence rate
ATG16L1	Autophagy related 16 like 1
ATI	Amylase-trypsin inhibitor
BDA	British Dietetic Association
BMI	Body mass index
CDAI	Crohn’s Disease Activity Index
CD	Crohn’s disease
CGM	Continuous glucose monitoring
CI	Confidence interval
CNS	Conserved non-coding sequence
COX-2	Cyclooxygenase-2
CRP	C-reactive protein
CDED	Crohn’s Disease Exclusion Diet
DALY	Disability-adjusted life year
DAMP	Damage-associated molecular pattern
DCT	Dietary clinical trial
DNA	Deoxyribonucleic acid
DNMT	DNA methyltransferase
DSS	Dextran sulfate sodium
EEN	Exclusive enteral nutrition
EGCG	Epigallocatechin-3-gallate
ERK	Extracellular signal-regulated kinase
EVOO	Extra virgin olive oil
FFQ	Food frequency questionnaire
FMT	Fecal microbiota transplantation
FODMAP	Fermentable oligosaccharides, disaccharides, monosaccharides, and polyols
Foxp3	Forkhead box P3
GBD	Global Burden of Disease
GI	Gastrointestinal
GIP	Gluten immunogenic peptides
GOS	Galactooligosaccharides
GPR	G protein-coupled receptor
GSH	Glutathione
GWAS	Genome-wide association study
H2RA	Histamine-2 receptor antagonist
HDAC	Histone deacetylase
HLA	Human leukocyte antigen
HR	Hazard ratio
IBS	Irritable bowel syndrome
IBS-C	IBS with constipation
IBS-D	IBS with diarrhea
IBS-M	IBS with mixed bowel habits
IBS-SSS	IBS Severity Scoring System
IBD	Inflammatory bowel disease
IFN-γ	Interferon-gamma
iHMP	Integrative Human Microbiome Project
IL	Interleukin
IRR	Incidence rate ratio
JNK	c-Jun N-terminal kinase
LPS	Lipopolysaccharide
MAPK	Mitogen-activated protein kinase
MD	Mediterranean diet
MDP	Muramyl dipeptide
mRNA	Messenger ribonucleic acid
MyD88	Myeloid differentiation primary response protein 88
NCGS	Non-celiac gluten sensitivity
NF-κB	Nuclear factor kappa-B
NLRP3	NOD-like receptor family pyrin domain containing 3
NOVA	Food processing classification system
NPH	Nutrition for Precision Health
NSAID	Nonsteroidal anti-inflammatory drug
OR	Odds ratio
PAMP	Pathogen-associated molecular pattern
PPAR-γ	Peroxisome proliferator-activated receptor gamma
PPI	Proton pump inhibitor
PUFA	Polyunsaturated fatty acid
QALY	Quality-adjusted life year
RCT	Randomized controlled trial
RNA	Ribonucleic acid
ROS	Reactive oxygen species
RR	Relative risk
SCFA	Short-chain fatty acid
SES-CD	Simple Endoscopic Score for Crohn’s Disease
SFA	Saturated fatty acid
SPM	Specialized pro-resolving mediator
TGF-β	Transforming growth factor-beta
Th	T helper cell
TLR	Toll-like receptor
TNBS	Trinitrobenzene sulfonic acid
TNF-α	Tumor necrosis factor-alpha
Treg	Regulatory T cell
UC	Ulcerative colitis
UI	Uncertainty interval
UPF	Ultra-processed food
ω-3	Omega-3 fatty acid

## References

[1] Wang Y., Huang Y., Chase R.C., Li T., Ramai D., Li S., Huang X., Antwi S.O., Keaveny A.P., Pang M. (2023). Global Burden of Digestive Diseases: A Systematic Analysis of the Global Burden of Diseases Study, 1990 to 2019. Gastroenterology.

[2] Abbafati C., Abbas K.M., Abbasi M., Abbasifard M., Abbasi-Kangevari M., Abbastabar H., Abd-Allah F., Abdelalim A., Abdollahi M., Abdollahpour I. (2020). Global Burden of 369 Diseases and Injuries in 204 Countries and Territories, 1990-2019: A Systematic Analysis for the Global Burden of Disease Study 2019. Lancet.

[3] Peery A.F., Crockett S.D., Murphy C.C., Jensen E.T., Kim H.P., Egberg M.D., Lund J.L., Moon A.M., Pate V., Barnes E.L. (2022). Burden and Cost of Gastrointestinal, Liver, and Pancreatic Diseases in the United States: Update 2021. Gastroenterology.

[4] Alatab S., Sepanlou S.G., Ikuta K., Vahedi H., Bisignano C., Safiri S., Sadeghi A., Nixon M.R., Abdoli A., Abolhassani H. (2020). The Global, Regional, and National Burden of Inflammatory Bowel Disease in 195 Countries and Territories, 1990-2017: A Systematic Analysis for the Global Burden of Disease Study 2017. Lancet Gastroenterol. Hepatol..

[5] Wang R., Li Z., Liu S., Zhang D. (2023). Global, Regional, and National Burden of 10 Digestive Diseases in 204 Countries and Territories from 1990 to 2019. Front. Public Health.

[6] Ross F.C., Patangia D., Grimaud G., Lavelle A., Dempsey E.M., Ross R.P., Stanton C. (2024). The Interplay between Diet and the Gut Microbiome: Implications for Health and Disease. Nat. Rev. Microbiol..

[7] Perler B.K., Friedman E.S., Wu G.D. (2023). The Role of the Gut Microbiota in the Relationship Between Diet and Human Health. Annu. Rev. Physiol..

[8] David L.A., Maurice C.F., Carmody R.N., Gootenberg D.B., Button J.E., Wolfe B.E., Ling A.V., Devlin A.S., Varma Y., Fischbach M.A. (2014). Diet Rapidly and Reproducibly Alters the Human Gut Microbiome. Nature.

[9] Singh R.K., Chang H.W., Yan D., Lee K.M., Ucmak D., Wong K., Abrouk M., Farahnik B., Nakamura M., Zhu T.H. (2017). Influence of Diet on the Gut Microbiome and Implications for Human Health. J. Transl. Med..

[10] Ghosh T.S., Rampelli S., Jeffery I.B., Santoro A., Neto M., Capri M., Giampieri E., Jennings A., Candela M., Turroni S. (2020). Mediterranean Diet Intervention Alters the Gut Microbiome in Older People Reducing Frailty and Improving Health Status: The NU-AGE 1-Year Dietary Intervention across Five European Countries. Gut.

[11] Lane M.M., Gamage E., Du S., Ashtree D.N., McGuinness A.J., Gauci S., Baker P., Lawrence M., Rebholz C.M., Srour B. (2024). Ultra-Processed Food Exposure and Adverse Health Outcomes: Umbrella Review of Epidemiological Meta-Analyses. BMJ.

[12] Vitale M., Costabile G., Testa R., D’Abbronzo G., Nettore I.C., Macchia P.E., Giacco R. (2024). Ultra-Processed Foods and Human Health: A Systematic Review and Meta-Analysis of Prospective Cohort Studies. Adv. Nutr..

[13] Dai S., Wellens J., Yang N., Li D., Wang J., Wang L., Yuan S., He Y., Song P., Munger R. (2024). Ultra-Processed Foods and Human Health: An Umbrella Review and Updated Meta-Analyses of Observational Evidence. Clin. Nutr..

[14] Narula N., Chang N.H., Mohammad D., Wong E.C.L., Ananthakrishnan A.N., Chan S.S.M., Carbonnel F., Meyer A. (2023). Food Processing and Risk of Inflammatory Bowel Disease: A Systematic Review and Meta-Analysis. Clin. Gastroenterol. Hepatol..

[15] Monteiro C.A., Cannon G., Levy R.B., Moubarac J.C., Louzada M.L.C., Rauber F., Khandpur N., Cediel G., Neri D., Martinez-Steele E. (2019). Ultra-Processed Foods: What They Are and How to Identify Them. Public Health Nutr..

[16] Khalili H., Håkansson N., Chan S.S., Chen Y., Lochhead P., Ludvigsson J.F., Chan A.T., Hart A.R., Olén O., Wolk A. (2020). Adherence to a Mediterranean Diet Is Associated with a Lower Risk of Later-Onset Crohn’s Disease: Results from Two Large Prospective Cohort Studies. Gut.

[17] Ratajczak A.E., Festa S., Aratari A., Papi C., Dobrowolska A., Krela-Kaźmierczak I. (2023). Should the Mediterranean Diet Be Recommended for Inflammatory Bowel Diseases Patients? A Narrative Review. Front. Nutr..

[18] Godny L., Dotan I. (2023). Is the Mediterranean Diet in Inflammatory Bowel Diseases Ready for Prime Time?. J. Can. Assoc. Gastroenterol..

[19] Lo C.H., Lochhead P., Khalili H., Song M., Tabung F.K., Burke K.E., Richter J.M., Giovannucci E.L., Chan A.T., Ananthakrishnan A.N. (2020). Dietary Inflammatory Potential and Risk of Crohn’s Disease and Ulcerative Colitis. Gastroenterology.

[20] Peters V., Bolte L., Schuttert E., Andreu-Sánchez S., Dijkstra G., Weersma R., Campmans-Kuijpers M. (2022). Western and Carnivorous Dietary Patterns Are Associated with Greater Likelihood of IBD Development in a Large Prospective Population-Based Cohort. J. Crohns Colitis.

[21] Limketkai B.N., Godoy-Brewer G., Parian A.M., Noorian S., Krishna M., Shah N.D., White J., Mullin G.E. (2023). Dietary Interventions for the Treatment of Inflammatory Bowel Diseases: An Updated Systematic Review and Meta-Analysis. Clin. Gastroenterol. Hepatol..

[22] Levine A., Wine E., Assa A., Sigall Boneh R., Shaoul R., Kori M., Cohen S., Peleg S., Shamaly H., On A. (2019). Crohn’s Disease Exclusion Diet Plus Partial Enteral Nutrition Induces Sustained Remission in a Randomized Controlled Trial. Gastroenterology.

[23] Lewis J.D., Sandler R.S., Brotherton C., Brensinger C., Li H., Kappelman M.D., Daniel S.G., Bittinger, K. C., Albenberg L., Valentine J.F. (2021). A Randomized Trial Comparing the Specific Carbohydrate Diet to a Mediterranean Diet in Adults with Crohn’s Disease. Gastroenterology.

[24] Bischoff S.C., Bager P., Escher J., Forbes A., Hébuterne X., Hvas C.L., Joly F., Klek S., Krznaric Z., Ockenga J. (2023). ESPEN Guideline on Clinical Nutrition in Inflammatory Bowel Disease. Clin. Nutr..

[25] Fitzpatrick J.A., Melton S.L., Yao C.K., Gibson P.R., Halmos E.P. (2022). Dietary Management of Adults with IBD—The Emerging Role of Dietary Therapy. Nat. Rev. Gastroenterol. Hepatol..

[26] Black C.J., Staudacher H.M., Ford A.C. (2022). Efficacy of a Low FODMAP Diet in Irritable Bowel Syndrome: Systematic Review and Network Meta-Analysis. Gut.

[27] van Lanen A.S., de Bree A., Greyling A. (2021). Efficacy of a Low-FODMAP Diet in Adult Irritable Bowel Syndrome: A Systematic Review and Meta-Analysis. Eur. J. Nutr..

[28] Dionne J., Ford A.C., Yuan Y., Chey W.D., Lacy B.E., Saito Y.A., Quigley E.M.M., Moayyedi P. (2018). A Systematic Review and Meta-Analysis Evaluating the Efficacy of a Gluten-Free Diet and a Low FODMAPs Diet in Treating Symptoms of Irritable Bowel Syndrome. Am. J. Gastroenterol..

[29] Staudacher H.M., Whelan K. (2017). The Low FODMAP Diet: Recent Advances in Understanding Its Mechanisms and Efficacy in IBS. Gut.

[30] Khalighi Sikaroudi M., Soltani S., Ghoreishy S.M., Ebrahimi Z., Shidfar F., Dehnad A. (2024). Effects of a Low FODMAP Diet on the Symptom Management of Patients with Irritable Bowel Syndrome: A Systematic Umbrella Review with the Meta-Analysis of Clinical Trials. Food Funct..

[31] Goodoory V.C., Khasawneh M., Black C.J., Quigley E.M.M., Moayyedi P., Ford A.C. (2023). Efficacy of Probiotics in Irritable Bowel Syndrome: Systematic Review and Meta-analysis. Gastroenterology.

[32] Zhang W.X., Shi L.B., Zhou M.S., Wu J., Shi H.Y. (2023). Efficacy of Probiotics, Prebiotics and Synbiotics in Irritable Bowel Syndrome: A Systematic Review and Meta-Analysis of Randomized, Double-Blind, Placebo-Controlled Trials. J. Med. Microbiol..

[33] Wu Y., Li Y., Zheng Q., Li L. (2024). The Efficacy of Probiotics, Prebiotics, Synbiotics, and Fecal Microbiota Transplantation in Irritable Bowel Syndrome: A Systematic Review and Network Meta-Analysis. Nutrients.

[34] Ford A.C., Harris L.A., Lacy B.E., Quigley E.M.M., Moayyedi P. (2018). Systematic Review with Meta-Analysis: The Efficacy of Prebiotics, Probiotics, Synbiotics and Antibiotics in Irritable Bowel Syndrome. Aliment. Pharmacol. Ther..

[35] Xie C.R., Tang B., Shi Y.Z., Peng W.Y., Ye K., Tao Q.F., Yu S.G., Zheng H., Chen M. (2022). Low FODMAP Diet and Probiotics in Irritable Bowel Syndrome: A Systematic Review with Network Meta-Analysis. Front. Pharmacol..

[36] King J.A., Jeong J., Underwood F.E., Quan J., Panaccione N., Windsor J.W., Coward S., Debruyn J., Ronksley P.E., Shaheen A.A. (2020). Incidence of Celiac Disease Is Increasing Over Time: A Systematic Review and Meta-Analysis. Am. J. Gastroenterol..

[37] Shiha M.G., Nandi N., Raju S.A., Wild G., Cross S.S., Singh P., Elli L., Makharia G.K., Sanders D.S., Penny H.A. (2024). Accuracy of the No-Biopsy Approach for the Diagnosis of Celiac Disease in Adults: A Systematic Review and Meta-Analysis. Gastroenterology.

[38] Silvester J.A., Kurada S., Szwajcer A., Kelly C.P., Leffler D.A., Duerksen D.R. (2017). Tests for Serum Transglutaminase and Endomysial Antibodies Do Not Detect Most Patients with Celiac Disease and Persistent Villous Atrophy on Gluten-Free Diets: A Meta-Analysis. Gastroenterology.

[39] Jadhav A., Bajaj A., Xiao Y., Markandey M., Ahuja V., Kashyap P.C. (2023). Role of Diet–Microbiome Interaction in Gastrointestinal Disorders and Strategies to Modulate Them with Microbiome-Targeted Therapies. Annu. Rev. Nutr..

[40] Huang P., Deng Y., Feng L., Gao Y., Cheng X., Liu H. (2023). Evaluation of Fetal Cardiac Function in Maternal Gestational Diabetes Mellitus by Speckle-Tracking Echocardiography. J. Ultrasound Med..

[41] Drago L., De La Motte L.R., Deflorio L., Sansico D.F., Salvatici M., Micaglio E., Biazzo M., Giarritiello F. (2025). Systematic Review of Bidirectional Interaction between Gut Microbiome, MiRNAs, and Human Pathologies. Front. Microbiol..

[42] Chauhan B., Dodamani S., Malik S., Almalki W.H., Haque S., Sayyed R.Z. (2013). Microbial Approaches for Pharmaceutical Wastewater Recycling and Management for Sustainable Development: A Multicomponent Approach. Environ. Res..

[43] Mazzola A.M., Zammarchi I., Valerii M.C., Spisni E., Saracino I.M., Lanzarotto F., Ricci C. (2024). Gluten-Free Diet and Other Celiac Disease Therapies: Current Understanding and Emerging Strategies. Nutrients.

[44] Rostami-Nejad M., Asri N., Olfatifar M., Khorsand B., Houri H., Rostami K. (2023). Systematic Review and Dose-Response Meta-Analysis on the Relationship between Different Gluten Doses and Risk of Coeliac Disease Relapse. Nutrients.

[45] Collinson S., Deans A., Padua-Zamora A., Gregorio G.V., Li C., Dans L.F., Allen S.J. (2020). Probiotics for Treating Acute Infectious Diarrhoea. Cochrane Database Syst. Rev..

[46] Derwa Y., Gracie D.J., Hamlin P.J., Ford A.C. (2017). Systematic Review with Meta-Analysis: The Efficacy of Probiotics in Inflammatory Bowel Disease. Aliment. Pharmacol. Ther..

[47] Kaur L., Gordon M., Baines P.A., Iheozor-Ejiofor Z., Sinopoulou V., Akobeng A.K. (2020). Probiotics for Induction of Remission in Ulcerative Colitis. Cochrane Database Syst. Rev..

[48] Iheozor-Ejiofor Z., Kaur L., Gordon M., Baines P.A., Sinopoulou V., Akobeng A.K. (2020). Probiotics for Maintenance of Remission in Ulcerative Colitis. Cochrane Database Syst. Rev..

[49] Vakadaris G., Stefanis C., Giorgi E., Brouvalis M., Voidarou C., Kourkoutas Y., Tsigalou C., Bezirtzoglou E. (2023). The Role of Probiotics in Inducing and Maintaining Remission in Crohn’s Disease and Ulcerative Colitis: A Systematic Review of the Literature. Biomedicines.

[50] Dalile B., Van Oudenhove L., Vervliet B., Verbeke K. (2019). The Role of Short-Chain Fatty Acids in Microbiota-Gut-Brain Communication. Nat. Rev. Gastroenterol. Hepatol..

[51] Parada Venegas D., De la Fuente M.K., Landskron G., González M.J., Quera R., Dijkstra G., Harmsen H.J.M., Faber K.N., Hermoso M.A. (2019). Short Chain Fatty Acids (SCFAs)-Mediated Gut Epithelial and Immune Regulation and Its Relevance for Inflammatory Bowel Diseases. Front. Immunol..

[52] Tan J., McKenzie C., Potamitis M., Thorburn A.N., Mackay C.R., Macia L. (2014). The Role of Short-Chain Fatty Acids in Health and Disease. Adv. Immunol..

[53] Koh A., De Vadder F., Kovatcheva-Datchary P., Bäckhed F. (2016). From Dietary Fiber to Host Physiology: Short-Chain Fatty Acids as Key Bacterial Metabolites. Cell.

[54] Louis P., Flint H.J. (2017). Formation of Propionate and Butyrate by the Human Colonic Microbiota. Environ. Microbiol..

[55] Aleksandrova K., Koelman L., Rodrigues C.E. (2021). Dietary Patterns and Biomarkers of Oxidative Stress and Inflammation: A Systematic Review of Observational and Intervention Studies. Redox Biol..

[56] Ding Y., Yanagi K., Yang F., Callaway E., Cheng C., Hensel M.E., Menon R., Alaniz R.C., Lee K., Jayaraman A. (2024). Oral Supplementation of Gut Microbial Metabolite Indole-3-Acetate Alleviates Diet-Induced Steatosis and Inflammation in Mice. Elife.

[57] Donohoe D.R., Garge N., Zhang X., Sun W., O’Connell T.M., Bunger M.K., Bultman S.J. (2011). The Microbiome and Butyrate Regulate Energy Metabolism and Autophagy in the Mammalian Colon. Cell Metab..

[58] Chang P.V., Hao L., Offermanns S., Medzhitov R. (2014). The Microbial Metabolite Butyrate Regulates Intestinal Macrophage Function via Histone Deacetylase Inhibition. Proc. Natl. Acad. Sci. USA.

[59] Tiffon C. (2018). The Impact of Nutrition and Environmental Epigenetics on Human Health and Disease. Int. J. Mol. Sci..

[60] Berry S.E., Valdes A.M., Drew D.A., Asnicar F., Mazidi M., Wolf J., Capdevila J., Hadjigeorgiou G., Davies R., Al Khatib H. (2020). Human Postprandial Responses to Food and Potential for Precision Nutrition. Nat. Med..

[61] Zeevi D., Korem T., Zmora N., Israeli D., Rothschild D., Weinberger A., Ben-Yacov O., Lador D., Avnit-Sagi T., Lotan-Pompan M. (2015). Personalized Nutrition by Prediction of Glycemic Responses. Cell.

[62] Kirk D., Catal C., Tekinerdogan B. (2021). Precision Nutrition: A Systematic Literature Review. Comput. Biol. Med..

[63] O’Sullivan A., Gibney M.J., Brennan L. (2011). Dietary Intake Patterns Are Reflected in Metabolomic Profiles: Potential Role in Dietary Assessment Studies. Am. J. Clin. Nutr..

[64] Menni C., Zierer J., Pallister T., Jackson M.A., Long T., Mohney R.P., Steves C.J., Spector T.D., Valdes A.M. (2017). Omega-3 Fatty Acids Correlate with Gut Microbiome Diversity and Production of N-Carbamylglutamate in Middle Aged and Elderly Women. Sci. Rep..

[65] Ford A.C., Sperber A.D., Corsetti M., Camilleri M. (2020). Irritable Bowel Syndrome. Lancet.

[66] Mayer E.A., Ryu H.J., Bhatt R.R. (2023). The Neurobiology of Irritable Bowel Syndrome. Mol. Psychiatry.

[67] Chey W.D., Keefer L., Whelan K., Gibson P.R. (2021). Behavioral and Diet Therapies in Integrated Care for Patients with Irritable Bowel Syndrome. Gastroenterology.

[68] Lacy B.E., Pimentel M., Brenner D.M., Chey W.D., Keefer L.A., Long M.D., Moshiree B. (2021). ACG Clinical Guideline: Management of Irritable Bowel Syndrome. Am. J. Gastroenterol..

[69] Böhn L., Störsrud S., Törnblom H., Bengtsson U., Simrén M. (2013). Self-Reported Food-Related Gastrointestinal Symptoms in IBS Are Common and Associated with More Severe Symptoms and Reduced Quality of Life. Am. J. Gastroenterol..

[70] Malfertheiner P., Megraud F., Rokkas T., Gisbert J.P., Liou J.M., Schulz C., Gasbarrini A., Hunt R.H., Leja M., O’Morain C. (2022). Management of *Helicobacter pylori* Infection: The Maastricht VI/Florence Consensus Report. Gut.

[71] Dore M.P., Bibbò S., Pes G.M., Francavilla R., Graham D.Y. (2019). Role of Probiotics in *Helicobacter pylori* Eradication: Lessons from a Study of *Lactobacillus reuteri* Strains DSM 17938 and ATCC PTA 6475 (Gastrus^®^) and a Proton-Pump Inhibitor. Can. J. Infect. Dis. Med. Microbiol..

[72] Fang H.R., Zhang G.Q., Cheng J.Y., Li Z.Y. (2019). Efficacy of Lactobacillus-Supplemented Triple Therapy for *Helicobacter pylori* Infection in Children: A Meta-Analysis of Randomized Controlled Trials. Eur. J. Pediatr..

[73] Ismail N.I., Nawawi K.N.M., Hsin D.C.C., Hao K.W., Mahmood N.R.K.N., Chearn G.L.C., Wong Z., Tamil A.M., Joseph H., Raja Ali R.A. (2023). Probiotic Containing *Lactobacillus reuteri* DSM 17648 as an Adjunct Treatment for *Helicobacter pylori* Infection: A Randomized, Double-Blind, Placebo-Controlled Trial. Helicobacter.

[74] Sakamoto I., Igarashi M., Kimura K., Takagi A., Miwa T., Koga Y. (2001). Suppressive Effect of *Lactobacillus gasseri* OLL 2716 (LG21) on *Helicobacter pylori* Infection in Humans. J. Antimicrob. Chemother..

[75] Narula N., Dhillon A., Zhang D., Sherlock M.E., Tondeur M., Zachos M. (2018). Enteral Nutritional Therapy for Induction of Remission in Crohn’s Disease. Cochrane Database Syst. Rev..

[76] Svolos V., Hansen R., Nichols B., Quince C., Ijaz U.Z., Papadopoulou R.T., Edwards C.A., Watson D., Alghamdi A., Brejnrod A. (2019). Treatment of Active Crohn’s Disease with an Ordinary Food-Based Diet That Replicates Exclusive Enteral Nutrition. Gastroenterology.

[77] Sigall-Boneh R., Pfeffer-Gik T., Segal I., Zangen T., Boaz M., Levine A. (2014). Partial Enteral Nutrition with a Crohn’s Disease Exclusion Diet Is Effective for Induction of Remission in Children and Young Adults with Crohn’s Disease. Inflamm. Bowel Dis..

[78] Ruemmele F.M., Veres G., Kolho K.L., Griffiths A., Levine A., Escher J.C., Amil Dias J., Barabino A., Braegger C.P., Bronsky J. (2014). Consensus Guidelines of ECCO/ESPGHAN on the Medical Management of Pediatric Crohn’s Disease. J. Crohns Colitis.

[79] Swaminath A., Feathers A., Ananthakrishnan A.N., Falzon L., Li Ferry S. (2017). Systematic Review with Meta-Analysis: Enteral Nutrition Therapy for the Induction of Remission in Paediatric Crohn’s Disease. Aliment. Pharmacol. Ther..

[80] Anguita-Ruiz A., Aguilera C.M., Gil Á. (2020). Genetics of Lactose Intolerance: An Updated Review and Online Interactive World Maps of Phenotype and Genotype Frequencies. Nutrients.

[81] Misselwitz B., Butter M., Verbeke K., Fox M.R. (2019). Update on Lactose Malabsorption and Intolerance: Pathogenesis, Diagnosis and Clinical Management. Gut.

[82] Oak S.J., Jha R. (2019). The Effects of Probiotics in Lactose Intolerance: A Systematic Review. Crit. Rev. Food Sci. Nutr..

[83] Savaiano D.A., Ritter A.J., Klaenhammer T.R., James G.M., Longcore A.T., Chandler J.R., Walker W.A., Foyt H.L. (2013). Improving Lactose Digestion and Symptoms of Lactose Intolerance with a Novel Galacto-Oligosaccharide (RP-G28): A Randomized, Double-Blind Clinical Trial. Nutr. J..

[84] He T., Priebe M.G., Zhong Y., Huang C., Harmsen H.J.M., Raangs G.C., Antoine J.M., Welling G.W., Vonk R.J. (2008). Effects of Yogurt and Bifidobacteria Supplementation on the Colonic Microbiota in Lactose-Intolerant Subjects. J. Appl. Microbiol..

[85] Reynolds A., Mann J., Cummings J., Winter N., Mete E., Te Morenga L. (2019). Carbohydrate Quality and Human Health: A Series of Systematic Reviews and Meta-Analyses. Lancet.

[86] Deng M., Dan L., Ye S., Chen X., Fu T., Wang X., Chen J. (2023). Higher Dietary Fibre Intake Is Associated with Lower Risk of Inflammatory Bowel Disease: Prospective Cohort Study. Aliment. Pharmacol. Ther..

[87] Swann O.G., Kilpatrick M., Breslin M., Oddy W.H. (2020). Dietary Fiber and Its Associations with Depression and Inflammation. Nutr. Rev..

[88] Sonnenburg J.L., Bäckhed F. (2016). Diet-Microbiota Interactions as Moderators of Human Metabolism. Nature.

[89] Holscher H.D. (2017). Dietary Fiber and Prebiotics and the Gastrointestinal Microbiota. Gut Microbes.

[90] Salminen S., Collado M.C., Endo A., Hill C., Lebeer S., Quigley E.M.M., Sanders M.E., Shamir R., Swann J.R., Szajewska H. (2021). The International Scientific Association of Probiotics and Prebiotics (ISAPP) Consensus Statement on the Definition and Scope of Postbiotics. Nat. Rev. Gastroenterol. Hepatol..

[91] Swanson K.S., Gibson G.R., Hutkins R., Reimer R.A., Reid G., Verbeke K., Scott K.P., Holscher H.D., Azad M.B., Delzenne N.M. (2020). The International Scientific Association for Probiotics and Prebiotics (ISAPP) Consensus Statement on the Definition and Scope of Synbiotics. Nat. Rev. Gastroenterol. Hepatol..

[92] Gibson G.R., Hutkins R., Sanders M.E., Prescott S.L., Reimer R.A., Salminen S.J., Scott K., Stanton C., Swanson K.S., Cani P.D. (2017). Expert Consensus Document: The International Scientific Association for Probiotics and Prebiotics (ISAPP) Consensus Statement on the Definition and Scope of Prebiotics. Nat. Rev. Gastroenterol. Hepatol..

[93] Hill C., Guarner F., Reid G., Gibson G.R., Merenstein D.J., Pot B., Morelli L., Canani R.B., Flint H.J., Salminen S. (2014). Expert Consensus Document. The International Scientific Association for Probiotics and Prebiotics Consensus Statement on the Scope and Appropriate Use of the Term Probiotic. Nat. Rev. Gastroenterol. Hepatol..

[94] Aguilar-Toalá J.E., Garcia-Varela R., Garcia H.S., Mata-Haro V., González-Córdova A.F., Vallejo-Cordoba B., Hernández-Mendoza A. (2018). Postbiotics: An Evolving Term within the Functional Foods Field. Trends Food Sci. Technol..

[95] Mimura T., Rizzello F., Helwig U., Poggioli G., Schreiber S., Talbot I.C., Nicholls R.J., Gionchetti P., Campieri M., Kamm M.A. (2004). Once Daily High Dose Probiotic Therapy (VSL#3) for Maintaining Remission in Recurrent or Refractory Pouchitis. Gut.

[96] Gionchetti P., Rizzello F., Venturi A., Brigidi P., Matteuzzi D., Bazzocchi G., Poggioli G., Miglioli M., Campieri M. (2000). Oral Bacteriotherapy as Maintenance Treatment in Patients with Chronic Pouchitis: A Double-Blind, Placebo-Controlled Trial. Gastroenterology.

[97] Whorwell P.J., Altringer L., Morel J., Bond Y., Charbonneau D., O’Mahony L., Kiely B., Shanahan F., Quigley E.M.M. (2006). Efficacy of an Encapsulated Probiotic *Bifidobacterium infantis* 35624 in Women with Irritable Bowel Syndrome. Am. J. Gastroenterol..

[98] Ducrotté P., Sawant P., Jayanthi V. (2012). Clinical Trial: *Lactobacillus plantarum* 299v (DSM 9843) Improves Symptoms of Irritable Bowel Syndrome. World J. Gastroenterol..

[99] Majeed M., Nagabhushanam K., Natarajan S., Sivakumar A., Ali F., Pande A., Majeed S., Karri S.K. (2016). *Bacillus coagulans* MTCC 5856 Supplementation in the Management of Diarrhea Predominant Irritable Bowel Syndrome: A Double Blind Randomized Placebo Controlled Pilot Clinical Study. Nutr. J..

[100] Cryan J.F., O’riordan K.J., Cowan C.S.M., Sandhu K.V., Bastiaanssen T.F.S., Boehme M., Codagnone M.G., Cussotto S., Fulling C., Golubeva A.V. (2019). The Microbiota-Gut-Brain Axis. Physiol. Rev..

[101] Margolis K.G., Cryan J.F., Mayer E.A. (2021). The Microbiota-Gut-Brain Axis: From Motility to Mood. Gastroenterology.

[102] Mayer E.A., Nance K., Chen S. (2022). The Gut-Brain Axis. Annu. Rev. Med..

[103] Collins S.M., Surette M., Bercik P. (2012). The Interplay between the Intestinal Microbiota and the Brain. Nat. Rev. Microbiol..

[104] Foster J.A., Rinaman L., Cryan J.F. (2017). Stress & the Gut-Brain Axis: Regulation by the Microbiome. Neurobiol. Stress.

[105] Farvid M.S., Sidahmed E., Spence N.D., Mante Angua K., Rosner B.A., Barnett J.B. (2021). Consumption of Red Meat and Processed Meat and Cancer Incidence: A Systematic Review and Meta-Analysis of Prospective Studies. Eur. J. Epidemiol..

[106] Di Y., Ding L., Gao L., Huang H. (2023). Association of Meat Consumption with the Risk of Gastrointestinal Cancers: A Systematic Review and Meta-Analysis. BMC Cancer.

[107] Chan D.S.M., Lau R., Aune D., Vieira R., Greenwood D.C., Kampman E., Norat T. (2011). Red and Processed Meat and Colorectal Cancer Incidence: Meta-Analysis of Prospective Studies. PLoS ONE.

[108] Poorolajal J., Mohammadi Y., Fattahi-Darghlou M., Almasi-Moghadam F. (2024). The Association between Major Gastrointestinal Cancers and Red and Processed Meat and Fish Consumption: A Systematic Review and Meta-Analysis of the Observational Studies. PLoS ONE.

[109] Huang Y., Cao D., Chen Z., Chen B., Li J., Guo J., Dong Q., Liu L., Wei Q. (2021). Red and Processed Meat Consumption and Cancer Outcomes: Umbrella Review. Food Chem..

[110] Ma G., Chen Y. (2020). Polyphenol Supplementation Benefits Human Health via Gut Microbiota: A Systematic Review via Meta-Analysis. J. Funct. Foods.

[111] Mao T., Zhang Y., Kaushik R., Mohan M.S. (2025). Effects of Polyphenols on Gut Microbiota and Inflammatory Markers in Individuals with Overweight or Obesity: A Systematic Review and Meta-Analysis of Randomized Controlled Trials. Crit. Rev. Food Sci. Nutr..

[112] Ozdal T., Sela D.A., Xiao J., Boyacioglu D., Chen F., Capanoglu E. (2016). The Reciprocal Interactions between Polyphenols and Gut Microbiota and Effects on Bioaccessibility. Nutrients.

[113] Cardona F., Andrés-Lacueva C., Tulipani S., Tinahones F.J., Queipo-Ortuño M.I. (2013). Benefits of Polyphenols on Gut Microbiota and Implications in Human Health. J. Nutr. Biochem..

[114] Bié J., Sepodes B., Fernandes P.C.B., Ribeiro M.H.L. (2023). Polyphenols in Health and Disease: Gut Microbiota, Bioaccessibility, and Bioavailability. Compounds.

[115] Younossi Z.M., Golabi P., Paik J.M., Henry A., Van Dongen C., Henry L. (2023). The Global Epidemiology of Nonalcoholic Fatty Liver Disease (NAFLD) and Nonalcoholic Steatohepatitis (NASH): A Systematic Review. Hepatology.

[116] Riazi K., Azhari H., Charette J.H., Underwood F.E., King J.A., Afshar E.E., Swain M.G., Congly S.E., Kaplan G.G., Shaheen A.A. (2022). The Prevalence and Incidence of NAFLD Worldwide: A Systematic Review and Meta-Analysis. Lancet Gastroenterol. Hepatol..

[117] Quek J., Chan K.E., Wong Z.Y., Tan C., Tan B., Lim W.H., Tan D.J.H., Tang A.S.P., Tay P., Xiao J. (2023). Global Prevalence of Non-Alcoholic Fatty Liver Disease and Non-Alcoholic Steatohepatitis in the Overweight and Obese Population: A Systematic Review and Meta-Analysis. Lancet Gastroenterol. Hepatol..

[118] Rinella M.E., Lazarus J.V., Ratziu V., Francque S.M., Sanyal A.J., Kanwal F., Romero D., Abdelmalek M.F., Anstee Q.M., Arab J.P. (2023). A Multisociety Delphi Consensus Statement on New Fatty Liver Disease Nomenclature. J. Hepatol..

[119] Xiong Y., Shi X., Xiong X., Li S., Zhao H., Song H., Wang J., Zhang L., You S., Ji G. (2024). A Systematic Review and Meta-Analysis of Randomized Controlled Trials: Effects of Mediterranean Diet and Low-Fat Diet on Liver Enzymes and Liver Fat Content of NAFLD. Food Funct..

[120] Targher G., Corey K.E., Byrne C.D., Roden M. (2021). The Complex Link between NAFLD and Type 2 Diabetes Mellitus—Mechanisms and Treatments. Nat. Rev. Gastroenterol. Hepatol..

[121] Suez J., Korem T., Zeevi D., Zilberman-Schapira G., Thaiss C.A., Maza O., Israeli D., Zmora N., Gilad S., Weinberger A. (2014). Artificial Sweeteners Induce Glucose Intolerance by Altering the Gut Microbiota. Nature.

[122] Suez J., Cohen Y., Valdés-Mas R., Mor U., Dori-Bachash M., Federici S., Zmora N., Leshem A., Heinemann M., Linevsky R. (2022). Personalized Microbiome-Driven Effects of Non-Nutritive Sweeteners on Human Glucose Tolerance. Cell.

[123] Conz A., Salmona M., Diomede L. (2023). Effect of Non-Nutritive Sweeteners on the Gut Microbiota. Nutrients.

[124] Gauthier E., Milagro F.I., Navas-Carretero S. (2024). Effect of Low-and Non-Calorie Sweeteners on the Gut Microbiota: A Review of Clinical Trials and Cross-Sectional Studies. Nutrition.

[125] Witkowski M., Nemet I., Alamri H., Wilcox J., Gupta N., Nimer N., Haghikia A., Li X.S., Wu Y., Saha P.P. (2023). The Artificial Sweetener Erythritol and Cardiovascular Event Risk. Nat. Med..

[126] Baunwall S.M.D., Lee M.M., Eriksen M.K., Mullish B.H., Marchesi J.R., Dahlerup J.F., Hvas C.L. (2020). Faecal Microbiota Transplantation for Recurrent *Clostridioides difficile* Infection: An Updated Systematic Review and Meta-Analysis. EClinicalMedicine.

[127] Tariq R., Syed T., Yadav D., Prokop L.J., Singh S., Loftus E.V., Pardi D.S., Khanna S. (2023). Outcomes of Fecal Microbiota Transplantation for *C. difficile* Infection in Inflammatory Bowel Disease: A Systematic Review and Meta-Analysis. J. Clin. Gastroenterol..

[128] Porcari S., Baunwall S.M.D., Occhionero A.S., Ingrosso M.R., Ford A.C., Hvas C.L., Gasbarrini A., Cammarota G., Ianiro G. (2023). Fecal Microbiota Transplantation for Recurrent *C. difficile* Infection in Patients with Inflammatory Bowel Disease: A Systematic Review and Meta-Analysis. J. Autoimmun..

[129] Tixier E.N., Verheyen E., Luo Y., Grinspan L.T., Du C.H., Ungaro R.C., Walsh S., Grinspan A.M. (2022). Systematic Review with Meta-Analysis: Fecal Microbiota Transplantation for Severe or Fulminant *Clostridioides difficile*. Dig. Dis. Sci..

[130] Willinger T. (2018). Oxysterols in Intestinal Immunity and Inflammation. J. Intern. Med..

[131] Hills R.D., Pontefract B.A., Mishcon H.R., Black C.A., Sutton S.C., Theberge C.R. (2019). Gut Microbiome: Profound Implications for Diet and Disease. Nutrients.

[132] Ahn J., Jun D.W., Lee H.Y., Moon J.H. (2019). Critical Appraisal for Low-Carbohydrate Diet in Nonalcoholic Fatty Liver Disease: Review and Meta-Analyses. Clin. Nutr..

[133] Eslam M., Sanyal A.J., George J., Sanyal A., Neuschwander-Tetri B., Tiribelli C., Kleiner D.E., Brunt E., Bugianesi E., Yki-Järvinen H. (2020). MAFLD: A Consensus-Driven Proposed Nomenclature for Metabolic Associated Fatty Liver Disease. Gastroenterology.

[134] Miao L., Targher G., Byrne C.D., Cao Y.Y., Zheng M.H. (2024). Current Status and Future Trends of the Global Burden of MASLD. Trends Endocrinol. Metab..

[135] Kelly C.R., Yen E.F., Grinspan A.M., Kahn S.A., Atreja A., Lewis J.D., Moore T.A., Rubin D.T., Kim A.M., Serra S. (2021). Fecal Microbiota Transplantation Is Highly Effective in Real-World Practice: Initial Results from the FMT National Registry. Gastroenterology.

[136] Bunyavanich S., Shen N., Grishin A., Wood R., Burks W., Dawson P., Jones S.M., Leung D.Y.M., Sampson H., Sicherer S. (2016). Early-Life Gut Microbiome Composition and Milk Allergy Resolution. J. Allergy Clin. Immunol..

[137] Canani R.B., Sangwan N., Stefka A.T., Nocerino R., Paparo L., Aitoro R., Calignano A., Khan A.A., Gilbert J.A., Nagler C.R. (2016). *Lactobacillus rhamnosus* GG-Supplemented Formula Expands Butyrate-Producing Bacterial Strains in Food Allergic Infants. ISME J..

[138] Tang M.L.K., Ponsonby A.L., Orsini F., Tey D., Robinson M., Su E.L., Licciardi P., Burks W., Donath S. (2015). Administration of a Probiotic with Peanut Oral Immunotherapy: A Randomized Trial. J. Allergy Clin. Immunol..

[139] Yu W., Freeland D.M.H., Nadeau K.C. (2016). Food Allergy: Immune Mechanisms, Diagnosis and Immunotherapy. Nat. Rev. Immunol..

[140] Spolidoro G.C.I., Amera Y.T., Ali M.M., Nyassi S., Lisik D., Ioannidou A., Rovner G., Khaleva E., Venter C., van Ree R. (2023). Frequency of Food Allergy in Europe: An Updated Systematic Review and Meta-Analysis. Allergy.

[141] Cabré E., Mañosa M., Gassull M.A. (2012). Omega-3 Fatty Acids and Inflammatory Bowel Diseases—A Systematic Review. Br. J. Nutr..

[142] Marton L.T., Goulart R.d.A., Carvalho A.C.A.d., Barbalho S.M. (2019). Omega Fatty Acids and Inflammatory Bowel Diseases: An Overview. Int. J. Mol. Sci..

[143] Astore C., Nagpal S., Gibson G. (2022). Mendelian Randomization Indicates a Causal Role for Omega-3 Fatty Acids in Inflammatory Bowel Disease. Int. J. Mol. Sci..

[144] Scaioli E., Sartini A., Bellanova M., Campieri M., Festi D., Bazzoli F., Belluzzi A. (2018). Eicosapentaenoic Acid Reduces Fecal Levels of Calprotectin and Prevents Relapse in Patients with Ulcerative Colitis. Clin. Gastroenterol. Hepatol..

[145] Ananthakrishnan A.N., Khalili H., Konijeti G.G., Higuchi L.M., De Silva P., Fuchs C.S., Willett W.C., Richter J.M., Chan A.T. (2014). Long-Term Intake of Dietary Fat and Risk of Ulcerative Colitis and Crohn’s Disease. Gut.

[146] Dziechciarz P., Horvath A., Shamir R., Szajewska H. (2007). Meta-Analysis: Enteral Nutrition in Active Crohn’s Disease in Children. Aliment. Pharmacol. Ther..

[147] Ding Z., Ninan K., Johnston B.C., Moayyedi P., Sherlock M., Zachos M. (2023). Microbiota Signatures and Mucosal Healing in the Use of Enteral Nutrition Therapy v. Corticosteroids for the Treatment of Children with Crohn’s Disease: A Systematic Review and Meta-Analysis. Br. J. Nutr..

[148] Yu Y., Chen K.C., Chen J. (2019). Exclusive Enteral Nutrition versus Corticosteroids for Treatment of Pediatric Crohn’s Disease: A Meta-Analysis. World J. Pediatr..

[149] Cena H., Calder P.C. (2020). Defining a Healthy Diet: Evidence for the Role of Contemporary Dietary Patterns in Health and Disease. Nutrients.

[150] Gaforio J.J., Visioli F., Alarcón-De-la-lastra C., Castañer O., Delgado-Rodríguez M., Fitó M., Hernández A.F., Huertas J.R., Martínez-González M.A., Menendez J.A. (2019). Virgin Olive Oil and Health: Summary of the III International Conference on Virgin Olive Oil and Health Consensus Report, JAEN (Spain) 2018. Nutrients.

[151] Coman L.I., Balaban D.V., Dumbravă B.F., Păunescu H., Marin R.C., Costescu M., Dima L., Jinga M., Coman O.A. (2025). Targeting Oxidative Stress in Acute Pancreatitis: A Critical Review of Antioxidant Strategies. Nutrients.

[152] Wang X., Qi Y., Zheng H. (2022). Dietary Polyphenol, Gut Microbiota, and Health Benefits. Antioxidants.

[153] Li H., Gao J., Peng W., Sun X., Qi W., Wang Y. (2025). Dietary Polyphenols-Gut Microbiota Interactions: Intervention Strategies and Metabolic Regulation for Intestinal Diseases. Biology.

[154] Hansen T., Duerksen D.R. (2018). Enteral Nutrition in the Management of Pediatric and Adult Crohn’s Disease. Nutrients.

[155] Muntean C., Blidari A.R., Faur A.M., Curca R.O., Feier C.V.I. (2024). Evaluating the Outcomes in Patients with Colorectal Cancer Using the Malnutrition Universal Screening Tool: A Systematic Review. J. Multidiscip. Healthc..

[156] Paukkonen I., Törrönen E.N., Lok J., Schwab U., El-Nezami H. (2024). The Impact of Intermittent Fasting on Gut Microbiota: A Systematic Review of Human Studies. Front. Nutr..

[157] Pérez-Gerdel T., Camargo M., Alvarado M., Ramírez J.D. (2023). Impact of Intermittent Fasting on the Gut Microbiota: A Systematic Review. Adv. Biol..

[158] Angoorani P., Ejtahed H.S., Hasani-Ranjbar S., Siadat S.D., Soroush A.R., Larijani B. (2021). Gut Microbiota Modulation as a Possible Mediating Mechanism for Fasting-Induced Alleviation of Metabolic Complications: A Systematic Review. Nutr. Metab..

[159] Guo Y., Luo S., Ye Y., Yin S., Fan J., Xia M. (2021). Intermittent Fasting Improves Cardiometabolic Risk Factors and Alters Gut Microbiota in Metabolic Syndrome Patients. J. Clin. Endocrinol. Metab..

[160] Su J., Wang Y., Zhang X., Ma M., Xie Z., Pan Q., Ma Z., Peppelenbosch M.P. (2021). Remodeling of the Gut Microbiome during Ramadan-Associated Intermittent Fasting. Am. J. Clin. Nutr..

[161] Dinu M., Pagliai G., Casini A., Sofi F. (2018). Mediterranean Diet and Multiple Health Outcomes: An Umbrella Review of Meta-Analyses of Observational Studies and Randomised Trials. Eur. J. Clin. Nutr..

[162] Estruch R., Ros E., Salas-Salvadó J., Covas M.-I., Corella D., Arós F., Gómez-Gracia E., Ruiz-Gutiérrez V., Fiol M., Lapetra J. (2018). Primary Prevention of Cardiovascular Disease with a Mediterranean Diet Supplemented with Extra-Virgin Olive Oil or Nuts. N. Engl. J. Med..

[163] Schwingshackl L., Hoffmann G. (2015). Diet Quality as Assessed by the Healthy Eating Index, the Alternate Healthy Eating Index, the Dietary Approaches to Stop Hypertension Score, and Health Outcomes: A Systematic Review and Meta-Analysis of Cohort Studies. J. Acad. Nutr. Diet..

[164] Mente A., De Koning L., Shannon H.S., Anand S.S. (2009). A Systematic Review of the Evidence Supporting a Causal Link between Dietary Factors and Coronary Heart Disease. Arch. Intern. Med..

[165] Afshin A., Sur P.J., Fay K.A., Cornaby L., Ferrara G., Salama J.S., Mullany E.C., Abate K.H., Abbafati C., Abebe Z. (2019). Health Effects of Dietary Risks in 195 Countries, 1990-2017: A Systematic Analysis for the Global Burden of Disease Study 2017. Lancet.

[166] Albillos A., de Gottardi A., Rescigno M. (2020). The Gut-Liver Axis in Liver Disease: Pathophysiological Basis for Therapy. J. Hepatol..

[167] Bajaj J.S., Heuman D.M., Hylemon P.B., Sanyal A.J., White M.B., Monteith P., Noble N.A., Unser A.B., Daita K., Fisher A.R. (2014). Altered Profile of Human Gut Microbiome Is Associated with Cirrhosis and Its Complications. J. Hepatol..

[168] Tripathi A., Debelius J., Brenner D.A., Karin M., Loomba R., Schnabl B., Knight R. (2018). Publisher Correction: The Gut–Liver Axis and the Intersection with the Microbiome. Nat. Rev. Gastroenterol. Hepatol..

[169] Tilg H., Cani P.D., Mayer E.A. (2016). Gut Microbiome and Liver Diseases. Gut.

[170] Pu X., Song Z., Han G. (2018). Competition among Supply Chains and Governmental Policy: Considering Consumers’ Low-Carbon Preference. Int. J. Environ. Res. Public Health.

[171] Ghasemi M., Etemadi A., Nedaei M., Chiniforush N., Pourhajibagher M. (2019). Antimicrobial Efficacy of Photodynamic Therapy Using Two Different Light Sources on the Titanium-Adherent Biofilms of *Aggregatibacter actinomycetemcomitans*: An in Vitro Study. Photodiagnosis Photodyn. Ther..

[172] Töröcsik D., Szanto A., Nagy L. (2009). Oxysterol Signaling Links Cholesterol Metabolism and Inflammation via the Liver X Receptor in Macrophages. Mol. Asp. Med..

[173] Fusano M., Fusano I., Galimberti M.G., Bencini M., Bencini P.L. (2020). Comparison of Postsurgical Scars Between Vegan and Omnivore Patients. Dermatol. Surg..

[174] Bungau A.F., Marin R.C., Tit D.M., Bungau G., Radu A., Branisteanu D.E., Endres L.M. (2025). Multifactorial Refractory Acne in Women: Insights from a Case Series Involving Hormonal-, Metabolic-, and Corticosteroid-Related Triggers. Life.

[175] Wahlström A., Sayin S.I., Marschall H.U., Bäckhed F. (2016). Intestinal Crosstalk between Bile Acids and Microbiota and Its Impact on Host Metabolism. Cell Metab..

[176] Ridlon J.M., Kang D.J., Hylemon P.B., Bajaj J.S. (2014). Bile Acids and the Gut Microbiome. Curr. Opin. Gastroenterol..

[177] Sayin S.I., Wahlström A., Felin J., Jäntti S., Marschall H.U., Bamberg K., Angelin B., Hyötyläinen T., Orešič M., Bäckhed F. (2013). Gut Microbiota Regulates Bile Acid Metabolism by Reducing the Levels of Tauro-Beta-Muricholic Acid, a Naturally Occurring FXR Antagonist. Cell Metab..

[178] Chiang J.Y.L., Ferrell J.M. (2018). Bile Acid Metabolism in Liver Pathobiology. Gene Expr..

[179] De Aguiar Vallim T.Q., Tarling E.J., Edwards P.A. (2013). Pleiotropic Roles of Bile Acids in Metabolism. Cell Metab..

[180] Dinan T.G., Cryan J.F. (2017). The Microbiome-Gut-Brain Axis in Health and Disease. Gastroenterol. Clin. N. Am..

[181] Valles-Colomer M., Falony G., Darzi Y., Tigchelaar E.F., Wang J., Tito R.Y., Schiweck C., Kurilshikov A., Joossens M., Wijmenga C. (2019). The Neuroactive Potential of the Human Gut Microbiota in Quality of Life and Depression. Nat. Microbiol..

[182] Kennedy P.J., Cryan J.F., Dinan T.G., Clarke G. (2017). Kynurenine Pathway Metabolism and the Microbiota-Gut-Brain Axis. Neuropharmacology.

[183] Yano J.M., Yu K., Donaldson G.P., Shastri G.G., Ann P., Ma L., Nagler C.R., Ismagilov R.F., Mazmanian S.K., Hsiao E.Y. (2015). Indigenous Bacteria from the Gut Microbiota Regulate Host Serotonin Biosynthesis. Cell.

[184] LeBlanc J.G., Milani C., de Giori G.S., Sesma F., van Sinderen D., Ventura M. (2013). Bacteria as Vitamin Suppliers to Their Host: A Gut Microbiota Perspective. Curr. Opin. Biotechnol..

[185] Magnúsdóttir S., Ravcheev D., De Crécy-Lagard V., Thiele I. (2015). Systematic Genome Assessment of B-Vitamin Biosynthesis Suggests Co-Operation among Gut Microbes. Front. Genet..

[186] Yoshii K., Hosomi K., Sawane K., Kunisawa J. (2019). Metabolism of Dietary and Microbial Vitamin B Family in the Regulation of Host Immunity. Front. Nutr..

[187] Degnan P.H., Taga M.E., Goodman A.L. (2014). Vitamin B12 as a Modulator of Gut Microbial Ecology. Cell Metab..

[188] Karl J.P., Meydani M., Barnett J.B., Vanegas S.M., Barger K., Fu X., Goldin B., Kane A., Rasmussen H., Vangay P. (2017). Fecal Concentrations of Bacterially Derived Vitamin K Forms Are Associated with Gut Microbiota Composition but Not Plasma or Fecal Cytokine Concentrations in Healthy Adults. Am. J. Clin. Nutr..

[189] Fasano A. (2011). Zonulin and Its Regulation of Intestinal Barrier Function: The Biological Door to Inflammation, Autoimmunity, and Cancer. Physiol. Rev..

[190] Bischoff S.C., Barbara G., Buurman W., Ockhuizen T., Schulzke J.D., Serino M., Tilg H., Watson A., Wells J.M. (2014). Intestinal Permeability—A New Target for Disease Prevention and Therapy. BMC Gastroenterol..

[191] Mu Q., Kirby J., Reilly C.M., Luo X.M. (2017). Leaky Gut As a Danger Signal for Autoimmune Diseases. Front. Immunol..

[192] Odenwald M.A., Turner J.R. (2017). The Intestinal Epithelial Barrier: A Therapeutic Target?. Nat. Rev. Gastroenterol. Hepatol..

[193] Camilleri M., Madsen K., Spiller R., Van Meerveld B.G., Verne G.N. (2012). Intestinal Barrier Function in Health and Gastrointestinal Disease. Neurogastroenterol. Motil..

[194] Birt D.F., Boylston T., Hendrich S., Jane J.L., Hollis J., Li L., McClelland J., Moore S., Phillips G.J., Rowling M. (2013). Resistant Starch: Promise for Improving Human Health. Adv. Nutr..

[195] Robertson M.D. (2012). Dietary-Resistant Starch and Glucose Metabolism. Curr. Opin. Clin. Nutr. Metab. Care.

[196] Maier T.V., Lucio M., Lee L.H., Verberkmoes N.C., Brislawn C.J., Bernhardt J., Lamendella R., McDermott J.E., Bergeron N., Heinzmann S.S. (2017). Impact of Dietary Resistant Starch on the Human Gut Microbiome, Metaproteome, and Metabolome. mBio.

[197] Bindels L.B., Walter J., Ramer-Tait A.E. (2015). Resistant Starches for the Management of Metabolic Diseases. Curr. Opin. Clin. Nutr. Metab. Care.

[198] Keenan M.J., Zhou J., Hegsted M., Pelkman C., Durham H.A., Coulon D.B., Martin R.J. (2015). Role of Resistant Starch in Improving Gut Health, Adiposity, and Insulin Resistance. Adv. Nutr..

[199] Turnbaugh P.J., Ley R.E., Mahowald M.A., Magrini V., Mardis E.R., Gordon J.I. (2006). An Obesity-Associated Gut Microbiome with Increased Capacity for Energy Harvest. Nature.

[200] Le Chatelier E., Nielsen T., Qin J., Prifti E., Hildebrand F., Falony G., Almeida M., Arumugam M., Batto J.M., Kennedy S. (2013). Richness of Human Gut Microbiome Correlates with Metabolic Markers. Nature.

[201] Cotillard A., Kennedy S.P., Kong L.C., Prifti E., Pons N., Le Chatelier E., Almeida M., Quinquis B., Levenez F., Galleron N. (2013). Dietary Intervention Impact on Gut Microbial Gene Richness. Nature.

[202] Ridaura V.K., Faith J.J., Rey F.E., Cheng J., Duncan A.E., Kau A.L., Griffin N.W., Lombard V., Henrissat B., Bain J.R. (2013). Gut Microbiota from Twins Discordant for Obesity Modulate Metabolism in Mice. Science.

[203] Dao M.C., Everard A., Aron-Wisnewsky J., Sokolovska N., Prifti E., Verger E.O., Kayser B.D., Levenez F., Chilloux J., Hoyles L. (2016). *Akkermansia muciniphila* and Improved Metabolic Health during a Dietary Intervention in Obesity: Relationship with Gut Microbiome Richness and Ecology. Gut.

[204] Depommier C., Everard A., Druart C., Plovier H., Van Hul M., Vieira-Silva S., Falony G., Raes J., Maiter D., Delzenne N.M. (2019). Supplementation with *Akkermansia muciniphila* in Overweight and Obese Human Volunteers: A Proof-of-Concept Exploratory Study. Nat. Med..

[205] Cani P.D., de Vos W.M. (2017). Next-Generation Beneficial Microbes: The Case of *Akkermansia muciniphila*. Front. Microbiol..

[206] Plovier H., Everard A., Druart C., Depommier C., Van Hul M., Geurts L., Chilloux J., Ottman N., Duparc T., Lichtenstein L. (2017). A Purified Membrane Protein from *Akkermansia muciniphila* or the Pasteurized Bacterium Improves Metabolism in Obese and Diabetic Mice. Nat. Med..

[207] Derrien M., Vaughan E.E., Plugge C.M., de Vos W.M. (2004). *Akkermansia muciniphila* Gen. Nov., Sp. Nov., a Human Intestinal Mucin-Degrading Bacterium. Int. J. Syst. Evol. Microbiol..

[208] Everard A., Belzer C., Geurts L., Ouwerkerk J.P., Druart C., Bindels L.B., Guiot Y., Derrien M., Muccioli G.G., Delzenne N.M. (2013). Cross-Talk between *Akkermansia muciniphila* and Intestinal Epithelium Controls Diet-Induced Obesity. Proc. Natl. Acad. Sci. USA.

[209] Blachier F., Davila A.M., Mimoun S., Benetti P.H., Atanasiu C., Andriamihaja M., Benamouzig R., Bouillaud F., Tomé D. (2010). Luminal Sulfide and Large Intestine Mucosa: Friend or Foe?. Amino Acids.

[210] Ijssennagger N., van der Meer R., van Mil S.W.C. (2016). Sulfide as a Mucus Barrier-Breaker in Inflammatory Bowel Disease?. Trends Mol. Med..

[211] Magee E.A., Richardson C.J., Hughes R., Cummings J.H. (2000). Contribution of Dietary Protein to Sulfide Production in the Large Intestine: An in Vitro and a Controlled Feeding Study in Humans. Am. J. Clin. Nutr..

[212] Attene-Ramos M.S., Wagner E.D., Plewa M.J., Gaskins H.R. (2006). Evidence That Hydrogen Sulfide Is a Genotoxic Agent. Mol. Cancer Res..

[213] Carbonero F., Benefiel A.C., Alizadeh-Ghamsari A.H., Gaskins H.R. (2012). Microbial Pathways in Colonic Sulfur Metabolism and Links with Health and Disease. Front. Physiol..

[214] Abeltino A., Hatem D., Serantoni C., Riente A., De Giulio M.M., De Spirito M., De Maio F., Maulucci G. (2024). Unraveling the Gut Microbiota: Implications for Precision Nutrition and Personalized Medicine. Nutrients.

[215] Liu Y.Y. (2025). Deep Learning for Microbiome-Informed Precision Nutrition. Natl. Sci. Rev..

[216] Bungau A.F., Tit D.M., Bungau S.G., Vesa C.M., Radu A.F., Marin R.C., Endres L.M., Moleriu L.C. (2024). Exploring the Metabolic and Endocrine Preconditioning Associated with Thyroid Disorders: Risk Assessment and Association with Acne Severity. Int. J. Mol. Sci..

[217] Feier C.V.I., Muntean C., Bolboacă S.D., Olariu S. (2024). Exploratory Evaluation of Pre-Treatment Inflammation Profiles in Patients with Colorectal Cancer. Diseases.

[218] Lagoumintzis G., Patrinos G.P. (2023). Triangulating Nutrigenomics, Metabolomics and Microbiomics toward Personalized Nutrition and Healthy Living. Hum. Genom..

[219] Johnson A.J., Vangay P., Al-Ghalith G.A., Hillmann B.M., Ward T.L., Shields-Cutler R.R., Kim A.D., Shmagel A.K., Syed A.N., Walter J. (2019). Daily Sampling Reveals Personalized Diet-Microbiome Associations in Humans. Cell Host Microbe.

[220] Sidhu S.R.K., Kok C.W., Kunasegaran T., Ramadas A. (2023). Effect of Plant-Based Diets on Gut Microbiota: A Systematic Review of Interventional Studies. Nutrients.

[221] Tomova A., Bukovsky I., Rembert E., Yonas W., Alwarith J., Barnard N.D., Kahleova H. (2019). The Effects of Vegetarian and Vegan Diets on Gut Microbiota. Front. Nutr..

[222] Fackelmann G., Manghi P., Carlino N., Heidrich V., Piccinno G., Ricci L., Piperni E., Arrè A., Bakker E., Creedon A.C. (2025). Gut Microbiome Signatures of Vegan, Vegetarian and Omnivore Diets and Associated Health Outcomes across 21,561 Individuals. Nat. Microbiol..

[223] Kim M.S., Hwang S.S., Park E.J., Bae J.W. (2013). Strict Vegetarian Diet Improves the Risk Factors Associated with Metabolic Diseases by Modulating Gut Microbiota and Reducing Intestinal Inflammation. Environ. Microbiol. Rep..

[224] Zhang C., Björkman A., Cai K., Liu G., Wang C., Li Y., Xia H., Sun L., Kristiansen K., Wang J. (2018). Impact of a 3-Months Vegetarian Diet on the Gut Microbiota and Immune Repertoire. Front. Immunol..

[225] Marco M.L., Heeney D., Binda S., Cifelli C.J., Cotter P.D., Foligné B., Gänzle M., Kort R., Pasin G., Pihlanto A. (2017). Health Benefits of Fermented Foods: Microbiota and Beyond. Curr. Opin. Biotechnol..

[226] Leeuwendaal N.K., Stanton C., O’toole P.W., Beresford T.P. (2022). Fermented Foods, Health and the Gut Microbiome. Nutrients.

[227] Wastyk H.C., Fragiadakis G.K., Perelman D., Dahan D., Merrill B.D., Yu F.B., Topf M., Gonzalez C.G., Van Treuren W., Han S. (2021). Gut-Microbiota-Targeted Diets Modulate Human Immune Status. Cell.

[228] Valentino V., Magliulo R., Farsi D., Cotter P.D., O’Sullivan O., Ercolini D., De Filippis F. (2024). Fermented Foods, Their Microbiome and Its Potential in Boosting Human Health. Microb. Biotechnol..

[229] Mukherjee A., Breselge S., Dimidi E., Marco M.L., Cotter P.D. (2024). Fermented Foods and Gastrointestinal Health: Underlying Mechanisms. Nat. Rev. Gastroenterol. Hepatol..

[230] Naimi S., Viennois E., Gewirtz A.T., Chassaing B. (2021). Direct Impact of Commonly Used Dietary Emulsifiers on Human Gut Microbiota. Microbiome.

[231] Bancil A.S., Sandall A.M., Rossi M., Chassaing B., Lindsay J.O., Whelan K. (2021). Food Additive Emulsifiers and Their Impact on Gut Microbiome, Permeability, and Inflammation: Mechanistic Insights in Inflammatory Bowel Disease. J. Crohns Colitis.

[232] Chassaing B., Koren O., Goodrich J.K., Poole A.C., Srinivasan S., Ley R.E., Gewirtz A.T. (2015). Dietary Emulsifiers Impact the Mouse Gut Microbiota Promoting Colitis and Metabolic Syndrome. Nature.

[233] Laudisi F., Stolfi C., Monteleone G. (2019). Impact of Food Additives on Gut Homeostasis. Nutrients.

[234] Whelan K., Bancil A.S., Lindsay J.O., Chassaing B. (2024). Ultra-Processed Foods and Food Additives in Gut Health and Disease. Nat. Rev. Gastroenterol. Hepatol..

[235] Lotti S., Dinu M., Colombini B., Amedei A., Sofi F. (2023). Circadian Rhythms, Gut Microbiota, and Diet: Possible Implications for Health. Nutr. Metab. Cardiovasc. Dis..

[236] Zeb F., Wu X., Chen L., Fatima S., Haq I.U., Chen A., Li M., Feng Q. (2020). Effect of Time-Restricted Feeding on Metabolic Risk and Circadian Rhythm Associated with Gut Microbiome in Healthy Males. Br. J. Nutr..

[237] Thaiss C.A., Zeevi D., Levy M., Zilberman-Schapira G., Suez J., Tengeler A.C., Abramson L., Katz M.N., Korem T., Zmora N. (2014). Transkingdom Control of Microbiota Diurnal Oscillations Promotes Metabolic Homeostasis. Cell.

[238] Reitmeier S., Kiessling S., Clavel T., List M., Almeida E.L., Ghosh T.S., Neuhaus K., Grallert H., Linseisen J., Skurk T. (2020). Arrhythmic Gut Microbiome Signatures Predict Risk of Type 2 Diabetes. Cell Host Microbe.

[239] Gutierrez Lopez D.E., Lashinger L.M., Weinstock G.M., Bray M.S. (2021). Circadian Rhythms and the Gut Microbiome Synchronize the Host’s Metabolic Response to Diet. Cell Metab..

[240] Routy B., Le Chatelier E., Derosa L., Duong C.P.M., Alou M.T., Daillère R., Fluckiger A., Messaoudene M., Rauber C., Roberti M.P. (2018). Gut Microbiome Influences Efficacy of PD-1-Based Immunotherapy against Epithelial Tumors. Science.

[241] Gopalakrishnan V., Spencer C.N., Nezi L., Reuben A., Andrews M.C., Karpinets T.V., Prieto P.A., Vicente D., Hoffman K., Wei S.C. (2018). Gut Microbiome Modulates Response to Anti-PD-1 Immunotherapy in Melanoma Patients. Science.

[242] Davar D., Dzutsev A.K., McCulloch J.A., Rodrigues R.R., Chauvin J.M., Morrison R.M., Deblasio R.N., Menna C., Ding Q., Pagliano O. (2021). Fecal Microbiota Transplant Overcomes Resistance to Anti-PD-1 Therapy in Melanoma Patients. Science.

[243] Spencer C.N., McQuade J.L., Gopalakrishnan V., McCulloch J.A., Vetizou M., Cogdill A.P., Wadud Khan M.A., Zhang X., White M.G., Peterson C.B. (2021). Dietary Fiber and Probiotics Influence the Gut Microbiome and Melanoma Immunotherapy Response. Science.

[244] Routy B., Lenehan J.G., Miller W.H., Jamal R., Messaoudene M., Daisley B.A., Hes C., Al K.F., Martinez-Gili L., Punčochář M. (2023). Fecal Microbiota Transplantation plus Anti-PD-1 Immunotherapy in Advanced Melanoma: A Phase I Trial. Nat. Med..

[245] Żółkiewicz J., Marzec A., Ruszczyński M., Feleszko W. (2020). Postbiotics-A Step Beyond Pre- and Probiotics. Nutrients.

[246] Gurunathan S., Thangaraj P., Kim J.H. (2023). Postbiotics: Functional Food Materials and Therapeutic Agents for Cancer, Diabetes, and Inflammatory Diseases. Foods.

[247] Mayorgas A., Dotti I., Salas A. (2021). Microbial Metabolites, Postbiotics, and Intestinal Epithelial Function. Mol. Nutr. Food Res..

[248] Kavita, Om H., Chand U., Kushawaha P.K. (2024). Postbiotics: An Alternative and Innovative Intervention for the Therapy of Inflammatory Bowel Disease. Microbiol. Res..

[249] Camilleri M. (2019). Leaky Gut: Mechanisms, Measurement and Clinical Implications in Humans. Gut.

[250] Pavel F.M., Bungau S.G., Tit D.M., Ghitea T.C., Marin R.C., Radu A.F., Moleriu R.D., Ilias T., Bustea C., Vesa C.M. (2023). Clinical Implications of Dietary Probiotic Supplement (Associated with L-Glutamine and Biotin) in Ulcerative Colitis Patients’ Body Composition and Quality of Life. Nutrients.

[251] Thevaranjan N., Puchta A., Schulz C., Naidoo A., Szamosi J.C., Verschoor C.P., Loukov D., Schenck L.P., Jury J., Foley K.P. (2017). Age-Associated Microbial Dysbiosis Promotes Intestinal Permeability, Systemic Inflammation, and Macrophage Dysfunction. Cell Host Microbe.

[252] Usuda H., Okamoto T., Wada K. (2021). Leaky Gut: Effect of Dietary Fiber and Fats on Microbiome and Intestinal Barrier. Int. J. Mol. Sci..

[253] Zheng D., Liwinski T., Elinav E. (2020). Interaction between Microbiota and Immunity in Health and Disease. Cell Res..

[254] Round J.L., Mazmanian S.K. (2009). The Gut Microbiota Shapes Intestinal Immune Responses during Health and Disease. Nat. Rev. Immunol..

[255] Belkaid Y., Hand T.W. (2014). Role of the Microbiota in Immunity and Inflammation. Cell.

[256] Hooper L.V., Littman D.R., Macpherson A.J. (2012). Interactions between the Microbiota and the Immune System. Science.

[257] Atarashi K., Tanoue T., Shima T., Imaoka A., Kuwahara T., Momose Y., Cheng G., Yamasaki S., Saito T., Ohba Y. (2011). Induction of Colonic Regulatory T Cells by Indigenous Clostridium Species. Science.

[258] Song M., Chan A.T., Sun J. (2020). Influence of the Gut Microbiome, Diet, and Environment on Risk of Colorectal Cancer. Gastroenterology.

[259] Tilg H., Adolph T.E., Gerner R.R., Moschen A.R. (2018). The Intestinal Microbiota in Colorectal Cancer. Cancer Cell.

[260] O’Keefe S.J.D., Li J.V., Lahti L., Ou J., Carbonero F., Mohammed K., Posma J.M., Kinross J., Wahl E., Ruder E. (2015). Fat, Fibre and Cancer Risk in African Americans and Rural Africans. Nat. Commun..

[261] Mehta R.S., Nishihara R., Cao Y., Song M., Mima K., Qian Z.R., Nowak J.A., Kosumi K., Hamada T., Masugi Y. (2017). Association of Dietary Patterns with Risk of Colorectal Cancer Subtypes Classified by *Fusobacterium nucleatum* in Tumor Tissue. JAMA Oncol..

[262] Murphy N., Moreno V., Hughes D.J., Vodicka L., Vodicka P., Aglago E.K., Gunter M.J., Jenab M. (2019). Lifestyle and Dietary Environmental Factors in Colorectal Cancer Susceptibility. Mol. Asp. Med..

[263] Fung T.C., Olson C.A., Hsiao E.Y. (2017). Interactions between the Microbiota, Immune and Nervous Systems in Health and Disease. Nat. Neurosci..

[264] Marin R.C., Ianculescu M., Costescu M., Mocanu V., Mihăescu A.G., Fulga I., Coman O.A. (2025). Integrated Medical and Digital Approaches to Enhance Post-Bariatric Surgery Care: A Prototype-Based Evaluation of the NutriMonitCare System in a Controlled Setting. Nutrients.

[265] Morais L.H., Schreiber H.L., Mazmanian S.K. (2021). The Gut Microbiota-Brain Axis in Behaviour and Brain Disorders. Nat. Rev. Microbiol..

